# Discovery of a new class of triazole based inhibitors of acetyl transferase KAT2A

**DOI:** 10.1080/14756366.2022.2097447

**Published:** 2022-07-25

**Authors:** Roberta Pacifico, Nunzio Del Gaudio, Guglielmo Bove, Lucia Altucci, Lydia Siragusa, Gabriele Cruciani, Menotti Ruvo, Rosa Bellavita, Paolo Grieco, Mauro F. A. Adamo

**Affiliations:** a Centre for Synthesis and Chemical Biology (CSCB), Department of Chemistry, Royal College of Surgeons in Ireland, Dublin, Ireland; b Department of precision medicine, University of Campania Luigi Vanvitelli, Naples, Italy; c Molecular Horizon Srl, Bettona, Italy; d Laboratory for Chemometrics and Molecular Modeling, Department of Chemistry, Biology, and Biotechnology, University of Perugia, Perugia, Italy; e Institute of Biostructures and Bioimaging, Consiglio Nazionale delle Ricerche, Naples, Italy; f Department of Pharmacy, School of Medicine, University of Naples ‘Federico II’, Naples, Italy

**Keywords:** *N*-pyridine triazoles, KAT2A inhibitors, virtual screening, acetyl transferases, anti-cancer

## Abstract

We have recently developed a new synthetic methodology that provided both *N*-aryl-5-hydroxytriazoles and *N*-pyridine-4-alkyl triazoles. A selection of these products was carried through virtual screening towards targets that are contemporary and validated for drug discovery and development. This study determined a number of potential structure target dyads of which *N*-pyridinium-4-carboxylic-5-alkyl triazole displayed the highest score specificity towards KAT2A. Binding affinity tests of abovementioned triazole and related analogs towards KAT2A confirmed the predictions of the *in-silico* assay. Finally, we have run *in vitro* inhibition assays of selected triazoles towards KAT2A; the ensemble of binding and inhibition assays delivered pyridyl-triazoles carboxylates as the prototype of a new class of inhibitors of KAT2A.

## 1. Introduction

1,2,3-triazoles are commonly recognised as important chemical scaffolds as well as efficient molecules in the area of medicinal chemistry^1^^,^^2^. Given their versatile behaviour in acting as both Lewis acids and bases^3^^,^^4^, triazoles have been used as a core structural motif in a huge variety of drug classes such as: antimicrobial^5^^,^^6^, anti-inflammatory^7^, antineoplastic^8^, antiviral^9^ ,antihypertensive^10^ or as immunomodulatory agents^11^.

**Scheme 1.**
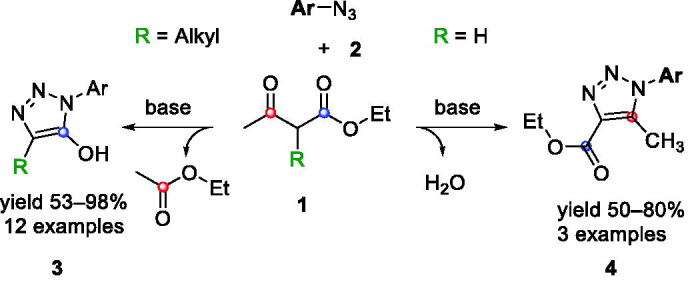
Preparation of triazoles **3** and **4**.

We have recently reported a novel synthetic pathway that, by reacting β-ketoesters **1** and azides **2**, provided 1,2,3-triazoles **3** or **4** (Scheme 1). The reactions employing 2-unsubstituted β-ketoesters were found to provide 5-methyl-1,2,3-triazoles **4**; whereas 2-alkyl-substituted β-ketoesters provided 5-hydroxy 1,2,3-triazoles **3** in high yields and as a single regioisomer^12^.

**Figure 1.**
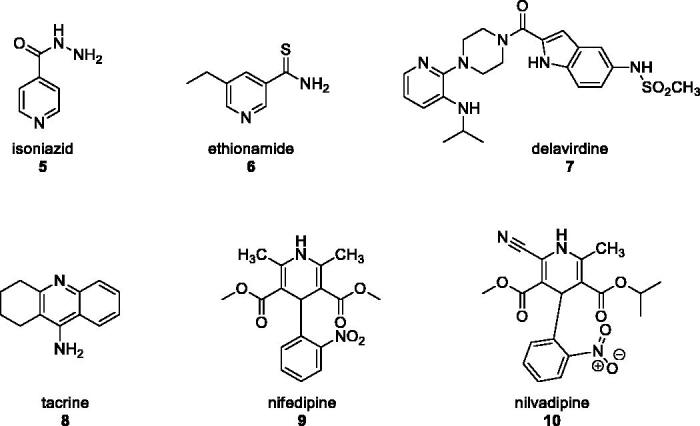
Few examples of approved drugs containing a pyridine unit.

As a follow up of this work, we have posed the question of whether or not those classes of new compounds may be useful in medicinal chemistry. Triazoles were repeatedly reported as bioactive compounds^1–11^ and many drug candidates containing the pyridine ring were equally described. Pyridines are commonly used in medicinal chemistry because of their ability to establish hydrogen bonds either as donors or acceptors, their water solubility, small dimensions and, most importantly, their potential to act as amide bioisosteres^13^. The latter in particular, makes pyridine pivotal in drug discovery^14^. Additionally, if compared to the benzene ring^15–17^, the pyridine unit displays a relevant increased basicity^18^, an improved aqueous solubility^19^ and a smaller polar surface; ^20^; all of these features consent an optimal orientation of the pyridine-containing drugs with the biological target through π-stacking interactions^21^^,^^22^. According to the FDA^23^, there are more than 95 approved drugs containing the pyridine moiety that are, currently, employed against tuberculosis (i.e., isoniazid^24^^,^^25^ **5** and ethionamide^26^ **6**), HIV/AIDS (i.e., delavirdine^27^ **7**), Alzheimer disease (i.e., tacrine^28^ **8**), Raynaud’s syndrome (i.e., nifedipine^29^ **9**), hypertension (e.g., nivaldipine^30^ **10**) and so on among the others (Figure 1).

**Figure 2.**
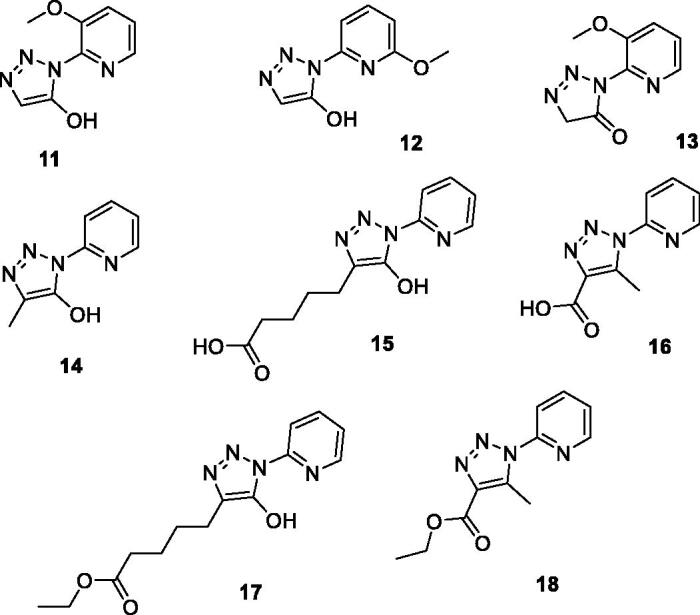
Pyridine-based triazoles tested through docking screening against the BioGPS cavity database.

**Figure 3.**
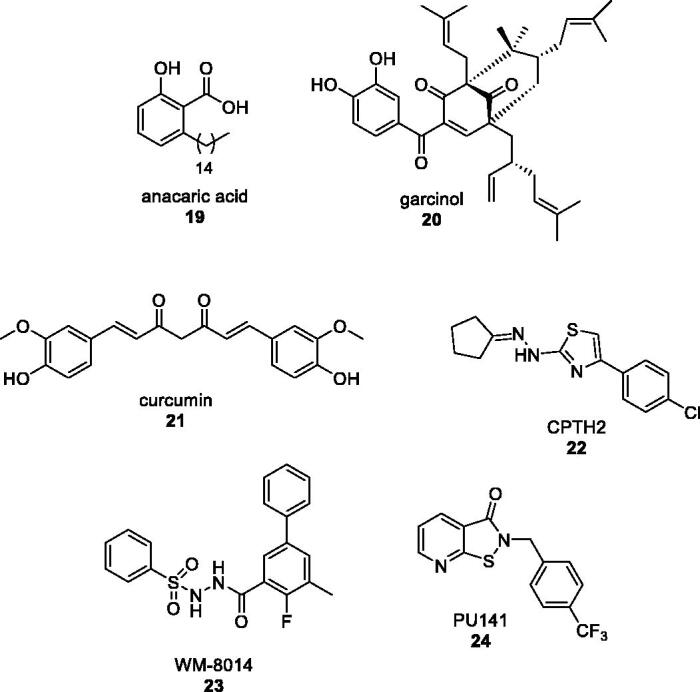
**N**atural and synthetic HATs inhibitors.

In order to answer the research question on new triazoles’ bioactivity, we submitted a small selection of pyridine-based compounds **11–18** (Figure 2) into a virtual screening study, which identified in *N*-(pyridin-2-yl)-4-carboxylic-5-methyl triazole **16** (Figure 2) a new chemical template with significant activity and selectivity as KAT2A inhibitor. KAT2A, also known as GCN5 (general control nonderepressible 5), is part of the HAT (histone acetyltransferase) family and, more specifically, belongs to the GCN5-related *N*-acetyltransferase group^31^^,^^32^. KAT2A promotes the acetylation of the ε-amino group of lysines thus preventing the positive charge on the amino group to impact other proteins interaction. Furthermore, the neutralisation of lysine positive charge leads to a weaker bond with DNA strands allowing transcriptional factors to penetrate DNA itself more easily^33^. Dysregulation of KATs’ activity is associated with numerous severe tumours such as small-cell lung cancer^34^ or colon cancer^35^, inflammatory disorders^36^, type 2 diabetes^37^ and so on. So far, only few HATs inhibitors are available and have been approved for therapeutical use (Figure 3). They mainly derive from natural sources such as anacardic acid^38^ **19**, garcinol^39^^,^^40^ **20** or curcumin^41^ **21** even though synthetic products, bearing thiazole^42^ **22**, isothiazole^43^^,^^44^ **23** and pyrido/benzothiazolone^45^ **24** units, have been recently introduced and proved to have a higher activity and specificity compared to the natural products.

Unfortunately, most of the HATs inhibitors known showed lack of selectivity towards members of the HAT family and, therefore, selectivity for a specific enzyme, for example, KAT2A, is still an outstanding issue.

Herein we describe each stage of our research that led to the identification of compound **16** (Figure 2) as a new KAT2A inhibitor template. This study includes virtual screening, synthesis, binding studies and *in vitro* testing.

## 2. Results and discussion

### 2.1. Virtual screening

Compounds **11–18** were submitted to virtual screening towards a database of proteins with established structure and role in system biology using the BioGPS software in combination with the FLAP algorithm. The BioGPS workflow used for the docking screening of triazoles **11–18** consisted in 5 parts: i) Protein refinement, achieved by using an algorithm known as “Fixpdb” that enables the preparation of the protein structure obtained from the Protein Data Bank (PDB)^46^; ii) Cavity detection, achieved by using an algorithm known as “Flapsite” that allows for the identification of pocket points located within a distance of 4 Å maximum from the closest protein^46^; iii) and iv) Cavity characterisation and comparison, achieved by the FLAP algorithm (Fingerprints for Ligands And Proteins) that enables the identification of the potential complementary ligand pharmacophoric features for a protein binding site^46^^,^^47^; v) Data analysis, in which each pocket/target similarity is analysed by using the global scores that attribute to 0 no similarity and to 1 maximum similarity^46^. This study provided docking results and score distribution of **11–18** versus the protein panel (Table 1)^46^. Importantly each of the proteins listed in Table 1 is a significant target for medicinal chemistry as for example KAT2A^31–37^.

**Table 1.**
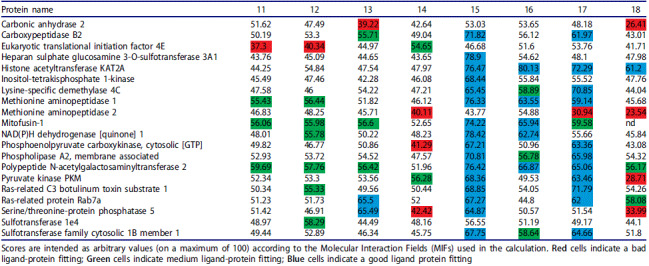
Docking scores for triazoles **11**–**18** found for this dataset of twenty proteins.

The results collated in Table 1 report the following: (1) numbers are arbitrary scores comprised between 1 and 100 and reflect the fitting of molecules **11–18** to the enzyme pocket; (2) red cells indicate a bad ligand-protein fitting; (3) green cells indicate medium ligand-protein fitting meanwhile (4) blue cells indicate a good ligand protein fitting. The results obtained showed the following: (i) triazoles **11–14**, bearing a hydroxyl group at position *C5*, did not show significant binding scores (Table 1, except for Rab7a^48^ and Serine/Threonine- protein phosphatase 5 (PP5)^49^; (ii) long chain acid **15** showed a good fitting with sixteen proteins (Table 1) hence lacked selectivity from the premises and for this reason was discarded. In conclusion, this dataset showed that the presence of a 5-hydroxy, or its ketol form, on the triazole generated a class of compounds that were not useful for further medicinal chemistry investigations with the 20 selected proteins. Equally, compound **17** (Table 1), showed a promiscuous behaviour with up to thirteen proteins and, therefore, was abandoned.

**Figure 4.**
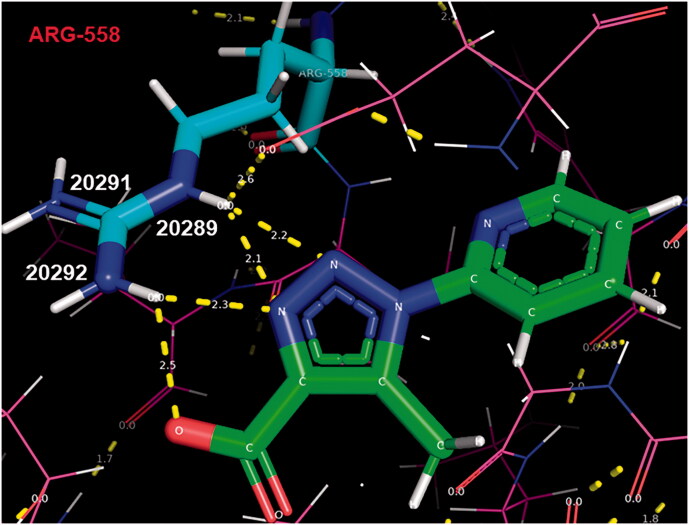
PyMOL outlook of triazole **16** bound to KAT2A active site pocket. Triazole’s **16** nitrogens are coloured in **blue**, carbons in **green** and oxygens in **red**.

At this point we focussed our attention on compound **16**, which docking with KAT2A was reported to be peculiarly higher than any of the other binders **11–15** and **17,18**, reaching 80.13 (Table 1)^1^. Considering that many of the known ligands for KAT2A comprise a carboxylate functionality (Figure 3), we hypothesised that the binding data for compound **16** were dependent on the presence of a carboxylic function at the *C4* position which favoured the interaction of triazole **16** with the desired target (Figure 4), whereas triazole **18**, bearing an ester function at *C4*, showed a lower binding score with KAT2A (Table 1). In support of our hypothesis, polar contacts connecting **16** and KAT2A occurred mainly when **16**’s OH function of the carboxylic moiety and **16**’s N2-N3 atoms of the triazole ring interacted with the NH functions of KAT2A Arg-558 residue (shown with blue and light blue sticks, Figure 4).

In particular, **16**’s N2 and N3 atoms of the triazole ring coordinate arginine NH number 20289 (2.2 Å and 2.1 Å distance respectively, Figure 4) while the N3 atom of the triazole ring and the OH function of the carboxylic moiety coordinate arginine NH number 20292 (2.3 Å and 2.5 Å distance respectively, Figure 4).

The low binding-affinity of triazole **18** with all the 20 proteins and the interactions of **16** with KAT2A identified in this work (Figure 4), clearly indicated that this selection of proteins required triazole binders to possess hydrogen-bond donor and acceptors units at *C4* and *C5*, which may have been a discriminator in the selection of targets from the BioGPS database^46^. In summary, the analysis of the docking studies identified **16** as a potential scaffold to be evaluated as KAT2A inhibitor. For this reason, we proceeded with the synthesis of a small library of triazole **16** analogs **26a-e** and **27a-d**.

### 2.2. Synthesis of pyridyl-triazoles 26a-f and 27a-d

A small library of 1,2,3-triazoles **26a**-**e** and **27a**-**d** bearing either a carboxylate or an ester functionality at *C4* was therefore prepared (Table 2). We anticipated that the carboxylic functionality was indeed crucial to high binding, however, we also noted that compound **18**, i.e., the ethyl ester of compound **16**, showed a significant binding for KAT2A. Hence, both carboxylates and their esters were included in the library.

**Table 2.** Synthesis of pyridyl-based triazoles **26a**-**e**^a^.

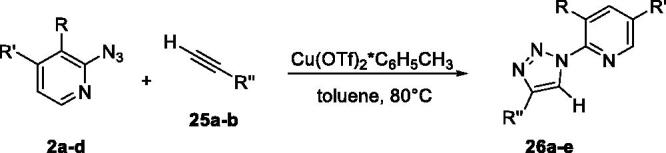							
Entry	Azide	R	R’	Alkyne	R’’	Product	Yield (%)^b^
1	**2a**	H	H	**25a**	COOEt	**26a**	90
2	**2a**	H	H	**25b**	(CH_2_)_4_COOH	**26b**	89
3	**2b**	OCH_3_	H	**25a**	COOEt	**26c**	78
4	**2c**	H	CH_3_	**25a**	COOEt	**26d**	94
5	**2d**	H	Cl	**25a**	COOEt	**26e**	99

^a^Reaction conditions: **2a**-**d** (1 equiv.), **25a**,**b** (1.1 equiv.), Cu(OTf)_2_*C_6_H_5_CH_3_ (0.1 equiv.), toluene (0.25 M). ^b^Isolated yields.

The synthesis of compounds **26a**-**e** has been carried out following a procedure previously reported^12^. Pyridine azides **2a**-**e** were reacted with alkynes **25a**,**b** to give triazoles **26a**-**e** *via* a CuAAc cycloaddition protocol (Table 2)^50^. Pyridine-based triazoles **26a**-**d** were obtained in high yields (Table 2). Considering that carboxylates were found better ligands by virtual screening, as highlighted in the docking results (Table 1), we then proceeded with the hydrolysis of esters **26a**,**b** and **26d,e** to reveal the corresponding acids **27a**-**d** (Table 3). This entailed standard saponification using a solution of potassium hydroxide, which provided **27a**-**d** in good to high yields.

**Table 3.** Hydrolysis of triazoles **26a,b-d,e** to reveal corresponding carboxylates **27a-d^a^**.

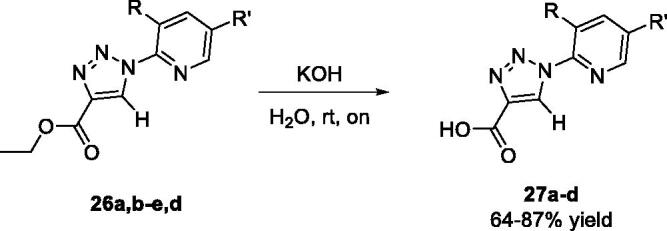					
Entry	Triazole	R	R’	Product	Yield (%)^b^
1	**26a**	H	H	**27a**	87
2	**26b**	OCH_3_	H	**27b**	70
3	**26d**	H	CH_3_	**27c**	71
4	**26e**	H	Cl	**27d**	64

^a^Reaction conditions: **26a,b**-**d**,**e** (1 equiv.), KOH (1 equiv.), H_2_O (1 M).

^b^Isolated yields.

### 2.3. Preliminary binding studies through fluorescence analysis

**Figure 5.**
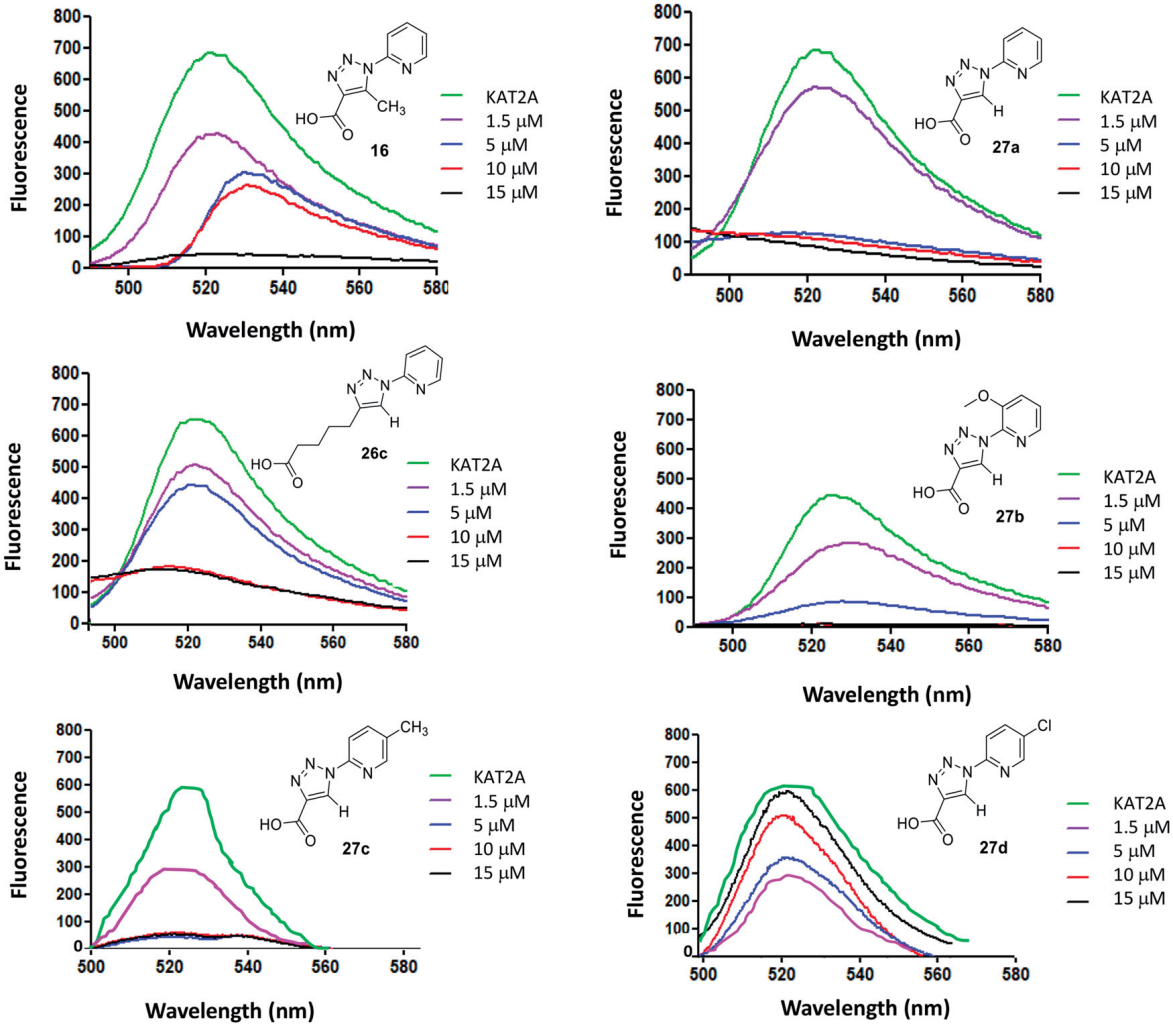
KAT2A fluorescence tests performed on triazoles **16**, **26c** and **27a**-**d**.

The binding properties, alongside the ability of **16** and **27a**-**d** to inhibit the activity of KAT2A were evaluated using a standard protocol based upon the measurement of fluorescence^51^. KAT2A fluorogenic assay was developed by others to screen for inhibitors of this enzyme^52^. This test is based on the transfer of an acetyl group from acetyl-CoA (acetyl coenzyme A) to a peptide substrate. After incubation with acetyl-CoA and the inhibitor, the KAT2A enzyme generates acetylated histone H3 peptide and CoASH. The thiol group of CoASH can be detected with fluorescein isothiocyanate isomer I (5-FITC), which is a reliable reaction considering the reactivity towards nucleophiles including amines and sulfhydryl groups present on proteins^51^. The fluorescein isothiocyanate isomer I (5-FITC) absorption is at wavelength = 495 nm and emission = 525 nm. In the experiment, which relies on the measurement of emission at 525 nm, strong emissions were observed when a poor inhibition of KAT2A was realised; on the contrary, poor emissions at 525 nm indicated high levels of inhibition (Figure 5). Each assay was run in triplicate and at increasing concentrations of **16**, **26c** and **27a**-**d** (1.5 µM, 5 µM, 10 µM and 15 µM). As shown in Figure 5, compounds **27a** and **27 b** displayed a good inhibitory activity at as low as 5 µM (coloured in blue) while higher concentrations were required for triazoles **16** and **26c** (15 µM, coloured in black, and 10 µM, coloured in red, respectively). Compound **27c** also displayed a good inhibition of KAT2A acetylating activity at just 5 µΜ concentration (coloured in blue). A significant divergence was observed for the behaviour of **27d**, which holds a *p*-Cl substitution on the pyridine ring. The results collected indicated that **27d** seemed to promote rather than inhibit the enzymatic activity of KAT2A, as highlighted by the increasing level of CoASH produced vs concentration (Figure 5). This result was most unexpected in lieu of the striking similarity between **27d** and **27a**-**c** and it should be looked in more details in future work.

In summary, we have demonstrated that triazoles **16**, **26c** and **27a-c** possess inhibitory activity of KAT2A whereas only **27d** proved to behave as an activator. Compounds **16**, **26c** and **27a**-**d** were then subjected to *in vitro* assays in a cell model displaying dysfunctional activity of KAT2A to confirm the bioactivity.

### 2.4. *In vitro* testing of triazoles 16, 26c and 27a-d

**Figure 6.**
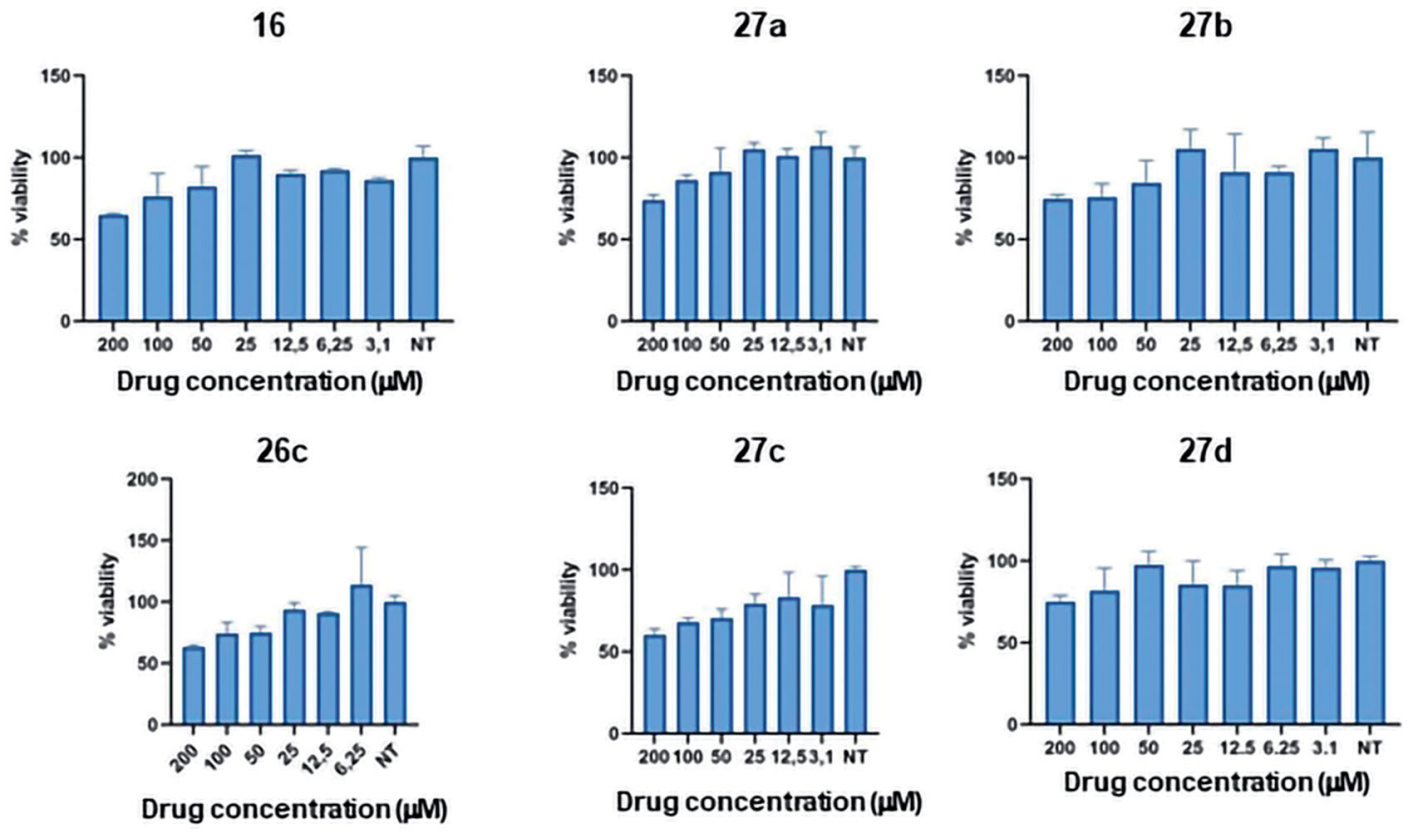
MTT assay performed on triazoles **16**, **26c** and **27a**-**d**. NT = non treated.

Compounds **16**, **26c** and **27a**-**d** were tested in U937 cell line (from human myeloid leukaemia, AML) in which KAT2A is known to be overexpressed^53^. U937 cells were stimulated with different concentrations of compounds **16**, **26c** and **27a**-**d**, starting from 200 µM. Cell viability was measured by thiazolyl blue tetrazolium bromide (MTT) assay. This assay displayed a slight reduction (10–20%) in cell viability by all the compounds tested (Figure 6).

**Figure 7.**
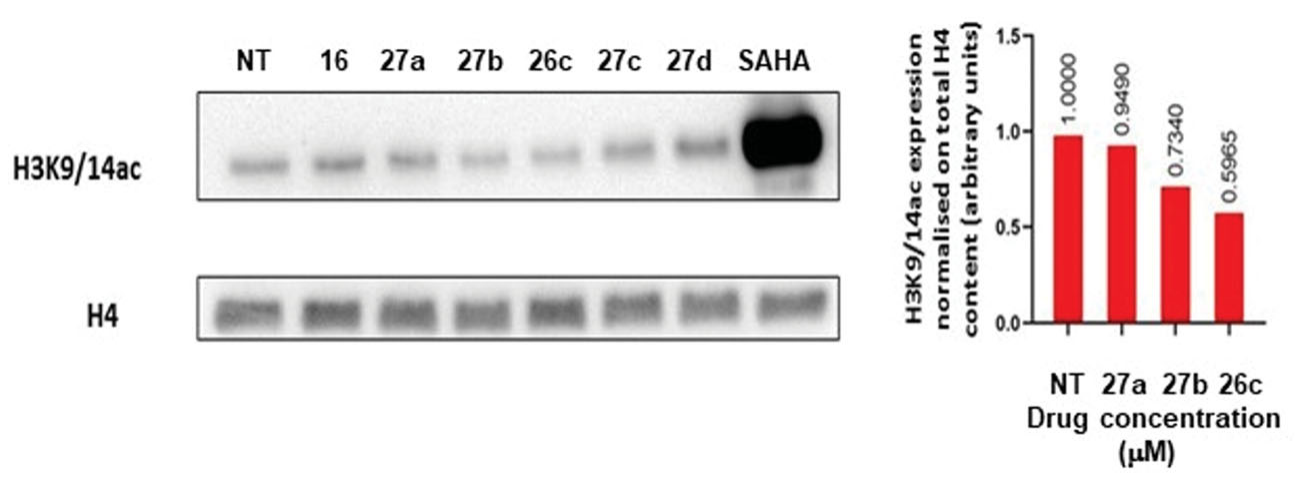
WB analysis (on the left) of **16**, **26c** and **27a-d** showing H3K9/14Ac levels in U937 cells following 24 h treatment at the concentration of 200 µM; 5 µM SAHA treatment was used as a positive control of acetylation. Densitometric analysis of WB is shown on the right.

To investigate compounds **16**, **26c** and **27a**-**d** induced inhibition of KAT2A acetylation levels of histone H3K9/14ac were analysed. To this end, U937 cells were treated with a 200 µM concentration of compounds **16**, **26c** and **27a**-**d** for 24 h. SAHA (suberoylanilide hydroxamic acid), a known histone deacetylase inhibitor, was used as a positive control of acetylation at 5 µM concentration. Histone extraction and subsequent Western Blot (WB) analysis were carried out checking H3K9/14ac acetylation levels. Triazole **26c** showed 40% reduction in the acetylation of H3K9/14ac (Figure 7) which was the strongest inhibition value obtained.

Considering the results obtained, it is possible that a lateral and flexible chain at *C4* on the triazole ring is required to allow a tight interaction of triazoles and KAT2A. This in addition to the presence of a hydrogen-bond acceptor site which is represented by the carboxylate function.

## 3. Conclusion

In conclusion, we identified a new class of binders/inhibitors of KAT2A which comprises a pyridine and a long chain carboxylate linked *via* a triazole ring. The study used virtual screening to select a small library of pyridine-based 1,2,3-triazoles, including **26a-e** and **27a-d**. We then submitted triazoles **26a-e** and **27a-d** to fluorescence binding assays versus KAT2A enzyme which confirmed the binding abilities of these entities to KAT2A. Fluorescence binding assays revealed that only triazoles **27a-d** and **26c** interacted with KAT2A, meanwhile their correspondent ester analogs **26a,b,e,f** did not show any binding. Finally, we evaluated the *in vitro* activity of **26c** and **27a-d** in U937 cell line of human AML and found out **26c** to be the most active compound showing a 40% inhibition of KAT2A acetylating activity. It is noteworthy that compound **26c**, having a longer chain, displayed the best inhibitory activity *in vitro*; meanwhile, shorter chain carboxylate, alike **16** or **27**, that were demonstrated optimal binders, showed reduced activity compared to **26c**. Studies are currently ongoing to (1) determine the structure-activity relationship (SAR) of **26c** analogs and (2) improve potency and selectivity of this new template (**26c**) for KAT2A inhibition at a lower dose concentration.

## Supplementary Material

Supplemental Material

## Note

1 Although the dataset predicted 16 as a good binder also for other proteins, the value of these interactions was inferior to a point that selectivity could be, at least in principle, possible.
