# Supplementary material for: Discovery of a new class of triazole based inhibitors of acetyl transferase KAT2A
Source: J Enzyme Inhib Med Chem. 2022 Jul 25;37(1):1987–94. doi: 10.1080/14756366.2022.2097447 (PMC9331200; doi:10.1080/14756366.2022.2097447)
Supplement: Supplemental Material [file IENZ_A_2097447_SM8844.pdf]

Electronic Supporting Information for the paper:

Discovery of a new class of triazole based inhibitors of acetyl transferase KAT2A

Roberta Pacifico[a], Nunzio Del Gaudio[b], Guglielmo Bove[b], Lucia Altucci[b], Lydia Siragusa[c], Gabriele Cruciani[d], Menotti Ruvo[e], Rosa Bellavita[f], Paolo Grieco[f], Mauro F. A. Adamo*[a].

Table of Contents

1. Experimental procedures S3

1.1 General procedure for the synthesis of pyridine-triazoles **26a-e** (GP1) S4

1.2 General procedure for the synthesis of hydrolysed pyridine-triazoles **27a-d** (GP2) S5

2. ^{1}H and ^{13}C copies of NMR spectra of triazoles **16**, **26a-e** and **27a-d** S6

3. *In vitro* tests performed on pyridyl-1,2,3-triazoles **16** and **27a-d** in U937 leukemia cell lines S17

3.1 Cell cultures S17

3.2 Western Blot analysis S17

3.3 Histones' extraction S17

3.4 Cell Proliferation Assay (MTT Assay) S18

3.5 Enhanced Cell Counting Kit 8 (WST-8/CCK8) S18

4. KAT2A assay performed on triazoles **16**, **26c** and **27a-d** S19

5. References S21

1. Experimental Procedures

^1H and ^{13}C NMR spectra were recorded on a Bruker 400 spectrometer. Chemical shifts (δ) are reported in ppm relative to residual solvent signals (^1H NMR: 7.26 ppm for $CDCl_3$; 3.31 ppm for CD_3OD, ^{13}C NMR: 77.16 ppm for $CDCl_3$, 49.03 for CD_3OD). ^{13}C NMR spectra were acquired with 1H broad band decoupled mode. Coupling constants (J) are in Hz. Melting points were measured using a Stuart scientific melting point apparatus and are uncorrected. Infrared spectra (IR) were recorded with KBr discs using a Bruker Tensor27 FT-IR instrument. High-resolution mass spectra were obtained on a Waters Micromass GCT PremierMS**.** Recombinant Human KAT2A/GCN5 His Protein from E.Coli source (corresponding to the amino acids 411-837 of Human KAT2A/GCN5) was purchased from Novus Biological. Acetyl CoA and H3 histone peptide were purchased from Sigma Aldrich. For the *in vitro* biological tests, fetal bovine serum (FBS) and SAHA were purchased from Sigma Aldrich, L-glutamine and antibiotics penicillin, streptomycin and amphotericin-B and U937 cell lines were purchased from Euroclone, KAT2A protease inhibitor cocktail was purchased from Roche, nitrocellulose membrane was purchased from Bio-Rad, α-tubulin was purchased from Cell Signal, antibodies anti-GADPH, anti-H4 and CCK8 assay kit were purchased from Elabscience and, finally, histones H3K9/14ac were purchased from Diagenode. Fluorescence of products **16**, **26c** and **27a-d** with KAT2A enzyme was measured on a Varian Cary Eclipse fluorescence spectrophotometer.

1.1 Docking Screening

The computational analysis was launched on the AWS E2 cloud (Amazon Web Service EC2) using the following virtual centralized processing unit (VCPU): 36 RAM: 72 G HD: SSD (GP2 type) on a Scalable 2nd Gen Intel Xeon (Cascade Lake) with an all-core turbo frequency of 3.6 GHz and a single-core turbo frequency up to 3.9 GHz. Docking screening of triazoles **11-18** has been performed testing each of the candidate compounds against a precomputed database of human crystallographic protein pockets (BioGPS pocketome).[2] The BioGPS workflow used for the docking screening of triazoles **11-18** consisted of 5 parts:

i) Protein refinement, achieved by using an algorithm known as "Fixpdb" that enables the preparation of the protein structure obtained from the Protein Data Bank (PDB). Additionally, Fixpdb also processes potential solvent molecules, co-crystallised ligands, cofactors or ions to be retained for consideration in subsequent analysis, if

considered essential for the protein functionality.[2]

ii) Cavity detection, achieved by using an algorithm known as "Flapsite" that embeds the protein structure into a tridimensional grid and allows for the identification of pocket points located only within a distance of 4 Å maximum from the closest protein atom using the GRID probe H, that discriminates according to the "shape" parameter.[2]

iii) Cavity characterisation, achieved by the FLAP program (Fingerprints for Ligands And Proteins) that enables the identification of the potential complementary ligand pharmacophoric features for a protein binding site. The GRID probes H, DRY, O and N1 are used to calculate the shape, the hydrophobic interactions and the H-bond acceptor and donor interactions respectively for each cavity considered in the analysis. Generally, the minimum numbers of energetically favorable interactions between a ligand and a protein is equal or greater than 4. FLAP then generates all possible combinations of four of the representative points returning them as quadruplets. All quadruplets generated for a specific cavity form the so called "Common Reference Framework".[2,3]

iv) Cavities comparison, occurred *via* comparison of binding sites via superimposition of the "Common Reference Framework" of the cavity against one or more template structures. A good match between cavity and template occurs when a pair of quadruplets have their distances coupled within 1 Å from each other.[2]

v) Data analysis, in which each final superimposition, called "solution", is identified by a set of Tanimoto similarity scores,[4] inteded as a measure of the similarity of two sets of elements obtained by dividing the intersection of the two sets over the union of the two sets. The global pocket-pocketsimilarity is analysed by using the global scores that attribute to 0 no similarity and to 1 maximum similarity.[2]

The 20 proteins identified by the screening have been chosen according to the best similarity score obtained for each protein pockets and the tested compounds. In particular, distribution scores of compounds **11-18** displayed a characteristic gaussian curve for each molecule that measured the probability the of these compounds to bind a protein's active site according to their

shape similarities. The global score of similarity values returned are represented by a number comprised between 0, meaning “no similarity”, and 1, meaning “maximum similarity”.

1.2 General procedure for the synthesis of pyridine-triazoles 26a-e (GP1)

To a solution of 2-azidopyridines **2a-d** (0.8 mmol, 1 equiv.) and copper triflate toluene complex (0.08 mmol, 0.1 equiv.) in dry toluene (0.25 M), alkynes **25a-b** (0.9 mmol, 1.1 equiv.) were added. The reaction mix stirred for 30min-24 hours at 80°C until complete consumption of the starting material, monitored by TLC (DCM/EtOAc 70:30). Once cooled to room temperature, the crude was washed with DCM/H_2O three times, the organic phases collected and dried over anhydrous sodium sulfate. Further purification occurred *via* column chromatography to afford the products **26a-e** (DCM/AcOEt 90:10) in good to excellent yields.

Ethyl 1-(pyridin-2-yl)-1H-1,2,3-triazole-4-carboxylate (26a)

Prepared according to GP1, yellow/orange solid (90%). ^{1}H NMR (400 MHz, $CDCl_3$) δ 9.10 (s, 1H), 8.55 (d, J = 4.7 Hz, 1H), 8.26 (d, J = 8.2 Hz, 1H), 7.97 (t, J = 7.8 Hz, 1H), 7.42 (dd, J = 7.3, 5.0 Hz, 1H), 4.48 (q, J = 7.1 Hz, 2H), 1.45 (t, J = 7.1 Hz, 3H). ^{13}C NMR (101 MHz, $CDCl_3$) δ 160.6, 148.8, 148.5, 140.5, 139.4, 124.8, 124.3, 114.2, 61.5, 14.3. All analytical data are consistent with those reported in the literature.[5,6]

Ethyl 1-(3-methoxypyridin-2-yl)-1H-1,2,3-triazole-4-carboxylate (26b)

Prepared according to GP1, yellowish solid (80%). ^{1}H NMR (400 MHz, $CDCl_3$) δ 8.73 (s, 1H), 8.26 (d, J = 4.4 Hz, 1H), 7.57 – 7.41 (m, 2H), 4.48 (q, J = 7.1 Hz, 2H), 2.93 (s, 3H), 1.45 (t, J = 7.1 Hz, 3H). ^{13}C NMR (101 MHz, $CDCl_3$) δ 160.9, 148.0, 140.5, 139.7, 138.1, 128.8, 126.0, 121.2, 61.5, 56.4, 14.4. IR (KBr, cm^{-1}): 2860, 1735, 1625, 1528, 1189. m.p. 100 °C. HRMS (ESI) m/z: $[M+H]^+$ calcd for $C_{11}H_{12}N_4O_3$ 248,2380; found: 249.1299.

5-(1-(pyridin-2-yl)-1H-1,2,3-triazol-4-yl)pentanoic acid (26c)

Prepared according to GP1, brown solid (70%). ^{1}H NMR (400 MHz, $CDCl_3$) δ 8.49 (d, J = 4.4 Hz, 1H), 8.35 (s, 1H), 8.19 (d, J = 8.2 Hz, 1H), 7.91 (t, J = 7.8 Hz, 1H), 7.36 – 7.31 (m, 1H), 2.86 (t, J = 7.1 Hz, 2H), 2.44 (t, J = 7.0 Hz, 2H), 1.89 – 1.71 (m, 4H). ^{13}C NMR (101 MHz, $CDCl_3$) δ 177.6, 149.3, 148.4, 139.1, 123.4, 118.4, 113.8, 28.6, 25.2, 24.2, 18.9. IR (KBr, cm^{-1}): 3149, 2880, 1726, 1635, 1566. m.p. 172 °C. HRMS (ESI) m/z: $[M+Na]^+$ calcd for $C_{12}H_{14}N_4O_2$ 246,1117; found: 269,1141.

Ethyl 1-(5-methylpyridin-2-yl)-1H-1,2,3-triazole-4-carboxylate (26d)

Prepared according to GP1, orange solid (94%). ^{1}H NMR (400 MHz, $CDCl_3$) δ 9.05 (s, 1H), 8.34 (s, 1H), 8.13 (d, J = 8.3 Hz, 1H), 7.75 (d, J = 8.2 Hz, 1H), 4.47 (q, J = 7.1 Hz, 2H), 2.44 (s, 3H), 1.44 (t, J = 7.1 Hz, 3H). ^{13}C NMR (101 MHz, $CDCl_3$) δ ^{13}C NMR (101 MHz, $CDCl_3$) δ 160.7, 148.8, 146.4, 140.4, 139.7, 134.4, 124.6, 113.6, 61.4, 18.1, 14.3. IR (KBr, cm^{-1}): 2860, 1724, 1645, 1466. m.p. 105 °C. HRMS (ESI) m/z: $[M+H]^+$ calcd for $C_{11}H_{12}N_4O_2$ 232,0960; found 233,2870.

Ethyl 1-(5-chloropyridin-2-yl)-1H-1,2,3-triazole-4-carboxylate (26e)

Prepared according to GP1, dark brown solid (99%). ^{1}H NMR (400 MHz, $CDCl_3$) δ 9.04 (s, 1H), 8.50 (s, 1H), 8.23 (d, J = 8.7 Hz, 1H), 7.94 (dd, J = 8.7, 2.1 Hz, 1H), 4.48 (q, J = 7.1 Hz, 2H), 1.44 (t, J = 7.1 Hz, 3H). ^{13}C NMR (101 MHz, $CDCl_3$) δ 160.4, 147.6, 146.7, 140.7, 139.1, 132.3, 124.8, 114.9, 61.6, 14.3. All analytical data are consistent with those reported in the literature.[7]

1.3 General procedure for the synthesis of hydrolised pyridine-triazoles 16 and 27a-d (GP2)

To a solution of triazoles **18** and **26a-e** (1 equiv., 0.2 mmol) in water (1 M), potassium hydroxide (1 equiv., 0.2 mmol) was added. The reaction mixture then stirred for 8 up to 24 hours at room temperature. Once TLC (DCM/MeOH 90:10) showed complete consumption of the starting triazoles, the crude was washed with ethyl acetate twice and the organic phases were discarded. The aqueous phases were collected and acidified with HCl 1 M (20 mL) and washed with ethyl acetate twice. The collected organic phases were dried over anhydrous sodium sulfate and the solvent was removed by rotary evaporation to afford title compounds **16** and **27a-d** in good yields and without further purification.

5-methyl-1-(pyridin-2-yl)-1H-1,2,3-triazole-4-carboxylic acid (16)

Prepared according to GP2, white solid (68%). ^{1}H NMR (400 MHz, $CDCl_3$) δ 8.62 (d, *J* = 4.5 Hz, 1H), 8.01-7.99 (m, 2H), 7.47 (t, *J* = 4.6 Hz, 1H), 2.96 (s, 3H). ^{13}C NMR (101 MHz, $CDCl_3$) δ 171.3, 150.1, 148.6, 140.6, 139.2, 136.7, 124.3, 118.3, 21.1. All analytical data are consistent with those reported in the literature.[8]

1-(pyridin-2-yl)-1H-1,2,3-triazole-4-carboxylic acid (27a)

Prepared according to GP2, white solid (87%). ^{1}H NMR (400 MHz, $CDCl_3$) δ 9.18 (s, 1H), 8.56 (d, J = 4.6 Hz, 1H), 8.26 (d, J = 8.2 Hz, 1H), 7.99 (t, J = 7.9 Hz, 1H), 7.44 (dd, J = 7.3, 5.1 Hz, 1H).^{13}C NMR (101 MHz, $CDCl_3$) δ 161.8, 148.9, 148.6, 140.5, 139.7, 124.8, 124.5, 113.8. All analytical data are consistent with those reported in the literature.[6]

1-(3-methoxypyridin-2-yl)-1H-1,2,3-triazole-4-carboxylic acid (27b)

Prepared according to GP2, orange solid (70%). ^{1}H NMR (400 MHz, MeOD) δ 8.93 (s, 1H), 8.20 (d, J = 4.9 Hz, 1H), 7.84 (d, J = 8.2 Hz, 1H), 7.64 (dd, J = 8.0, 4.5 Hz, 1H), 3.98 (s, 3H). ^{13}C NMR (101 MHz, MeOD) δ 162.0, 148.8, 139.7, 137.7, 129.3, 126.8, 122.0, 55.6, 53.4. IR (KBr, cm^{-1}): 3268, 2860, 1625, 1528, 1189. m.p. 160°C. HRMS (ESI) m/z: $[M+H]^+$ calcd for $C_9H_8N_4O_3$ 220,1848; found 221,2332.

1-(5-methylpyridin-2-yl)-1H-1,2,3-triazole-4-carboxylic acid (27c)

Prepared according to GP2, white solid (71%). ^{1}H NMR (400 MHz, MeOD) δ 9.15 (s, 1H), 8.42 (s, 1H), 8.08 (d, J = 8.3 Hz, 1H), 7.91 (d, J = 8.1 Hz, 1H), 2.44 (s, 3H). ^{13}C NMR (101 MHz, MeOD) δ 161.8, 148.8, 146.4, 140.3, 139.9, 135.1, 124.7, 113.3, 16.6. IR (KBr, cm^{-1}): 3256, 2860, 1645, 1466. m.p. 125 °C. HRMS (ESI) m/z: $[M+H]^+$ calcd for $C_9H_8N_4O_2$ 204,0647; found 205,1057.

1-(5-chloropyridin-2-yl)-1H-1,2,3-triazole-4-carboxylic acid (27d)

Prepared according to GP2, yellowish solid (64%). ^{1}H NMR (400 MHz, MeOD) δ 9.19 (s, 1H), 8.60 (s, 1H), 8.22 (d, J = 8.7 Hz, 1H), 8.14 (d, J = 8.7 Hz, 1H). ^{13}C NMR (101 MHz, MeOD) δ 161.6, 147.5, 146.9, 140.5, 139.4, 132.2, 125.0, 114.9. All analytical data are consistent with those reported in the literature.[6]

2. Copies of ^{1}H and ^{13}C NMR spectra

Compound **26a** (400 MHz, $CDCl_3$)

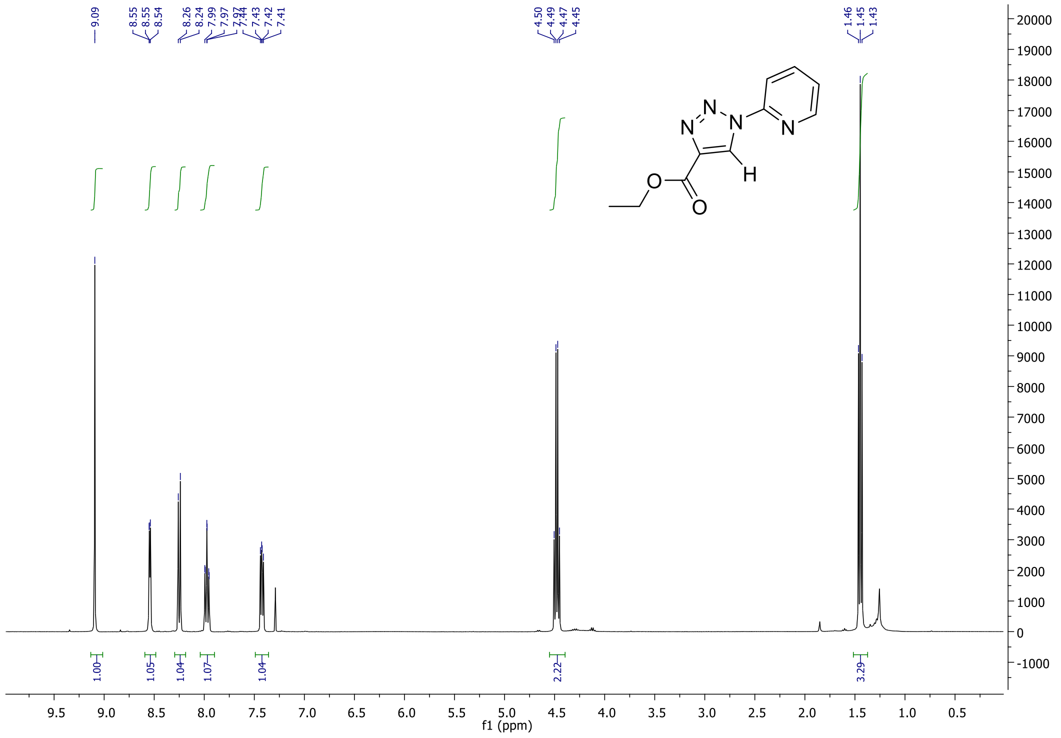

Compound **26a** ^{13}C NMR (101 MHz, $CDCl_3$)

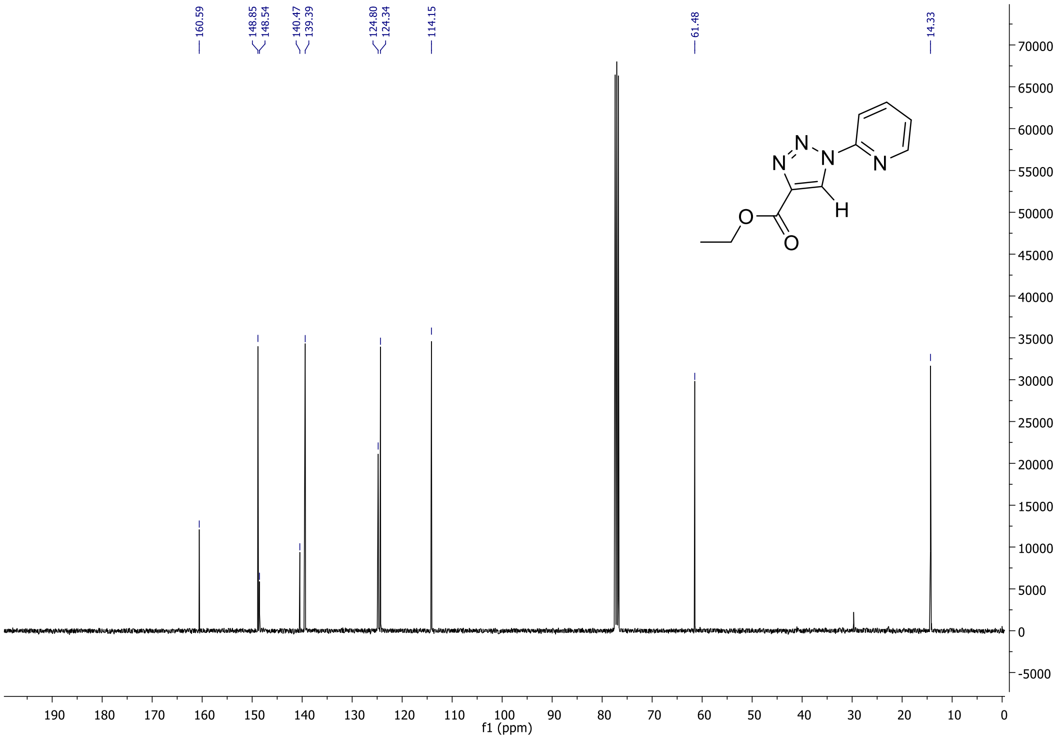

Compound **26b** (400 MHz, $CDCl_3$)

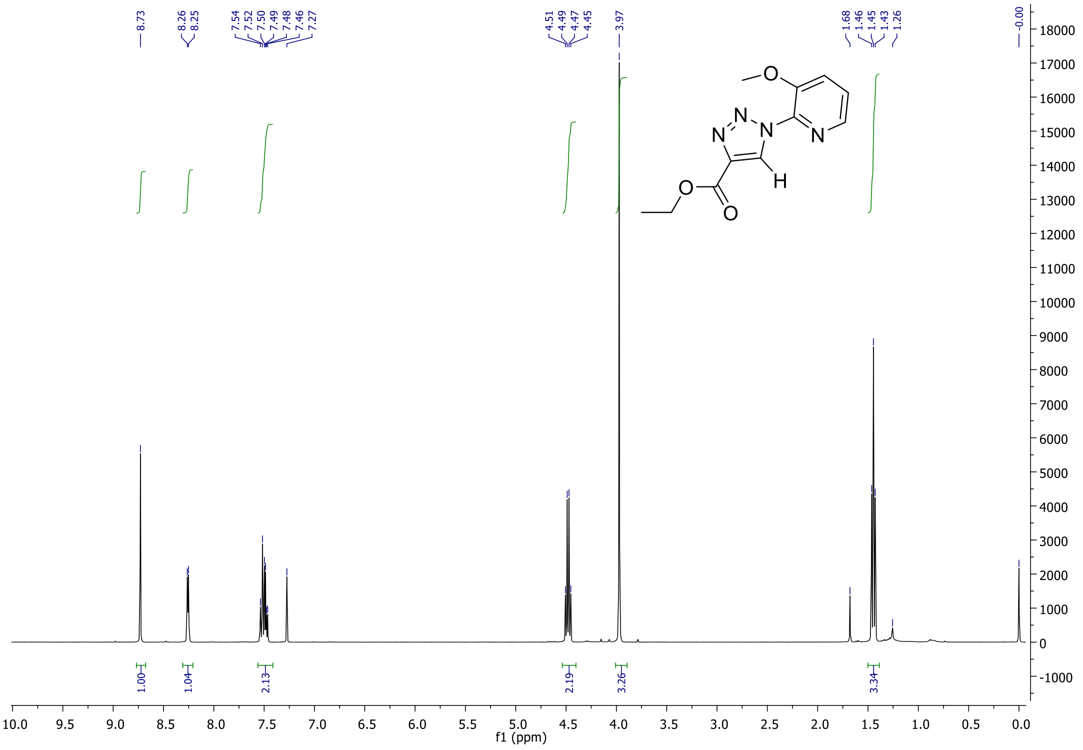

Compound **26b** ^{13}C NMR (101 MHz, $CDCl_3$)

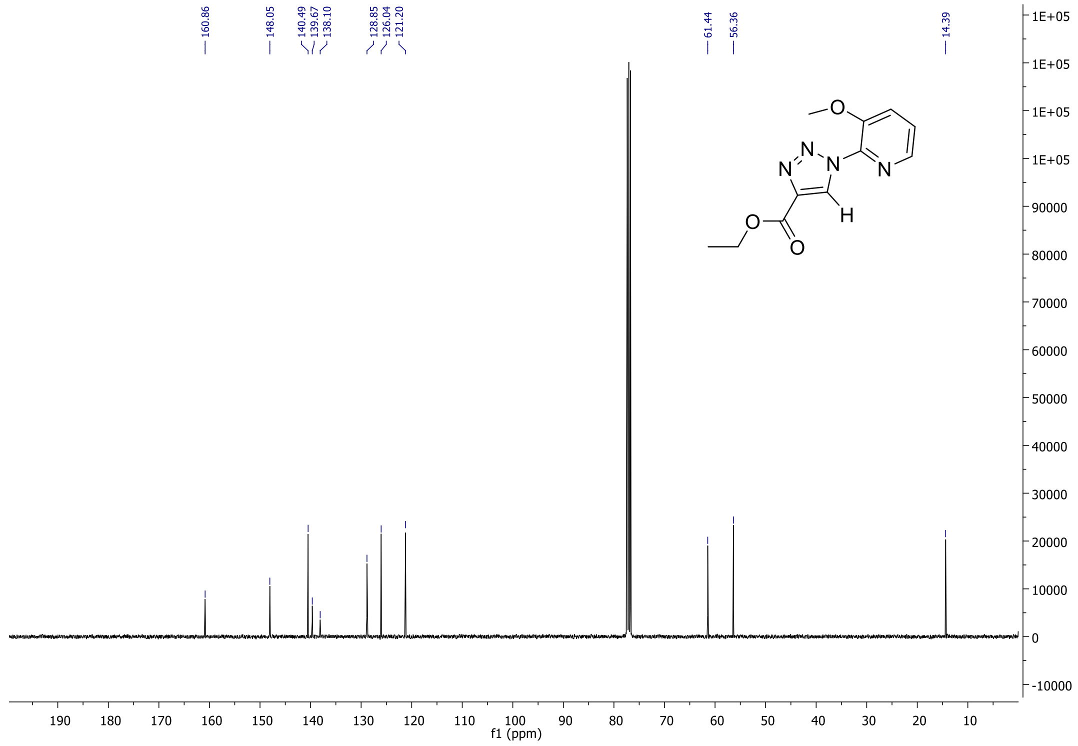

Compound **26c** (400 MHz, $CDCl_3$)

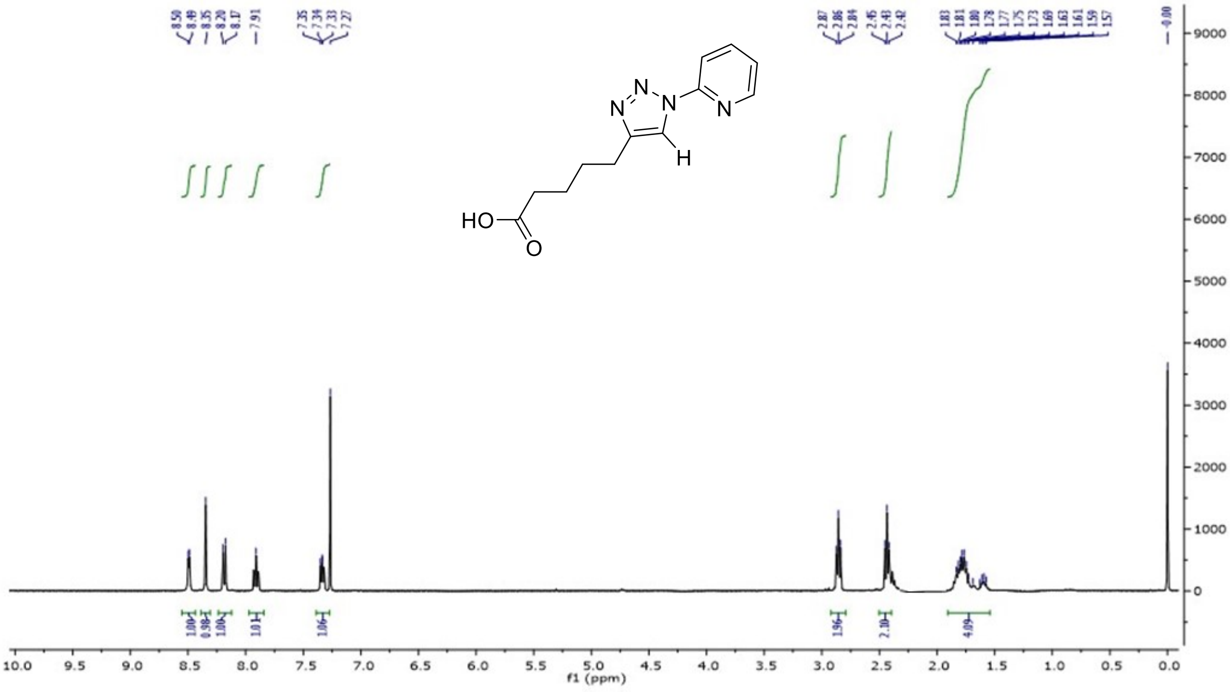

Compound **26c** ^{13}C NMR (101 MHz, $CDCl_3$)

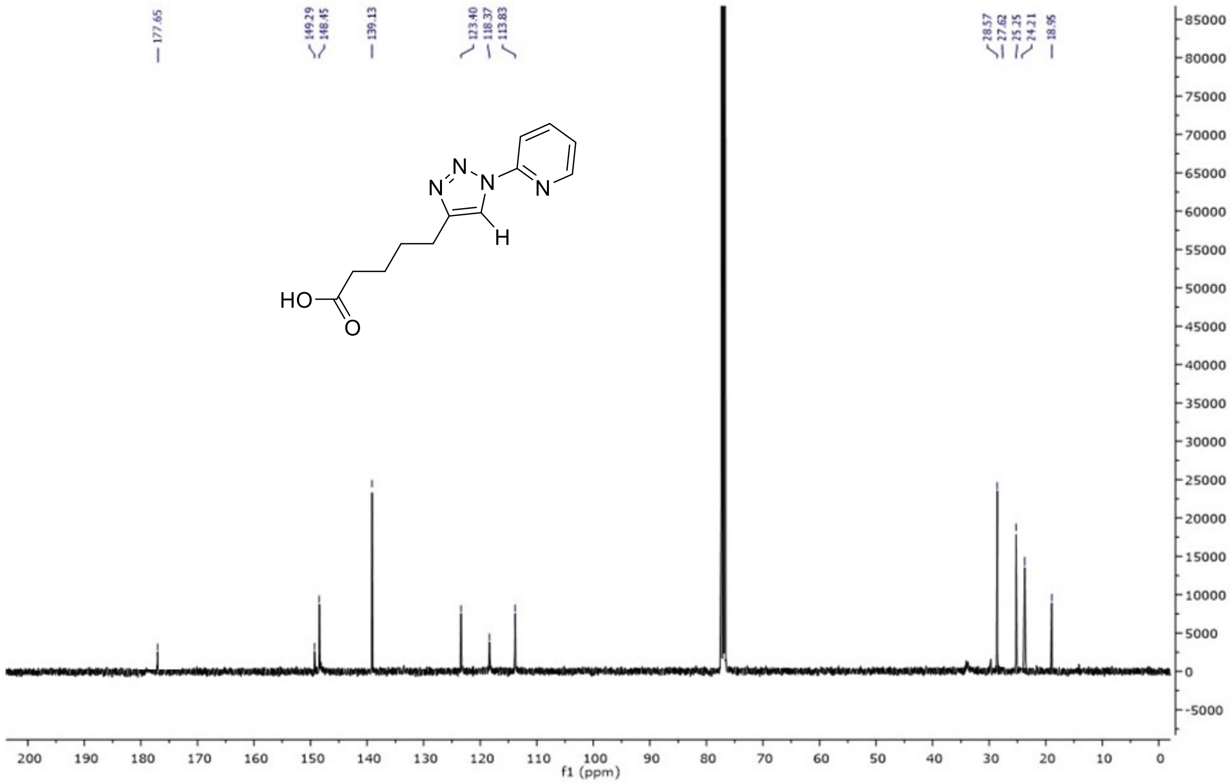

Compound **26d** (400 MHz, $CDCl_3$)

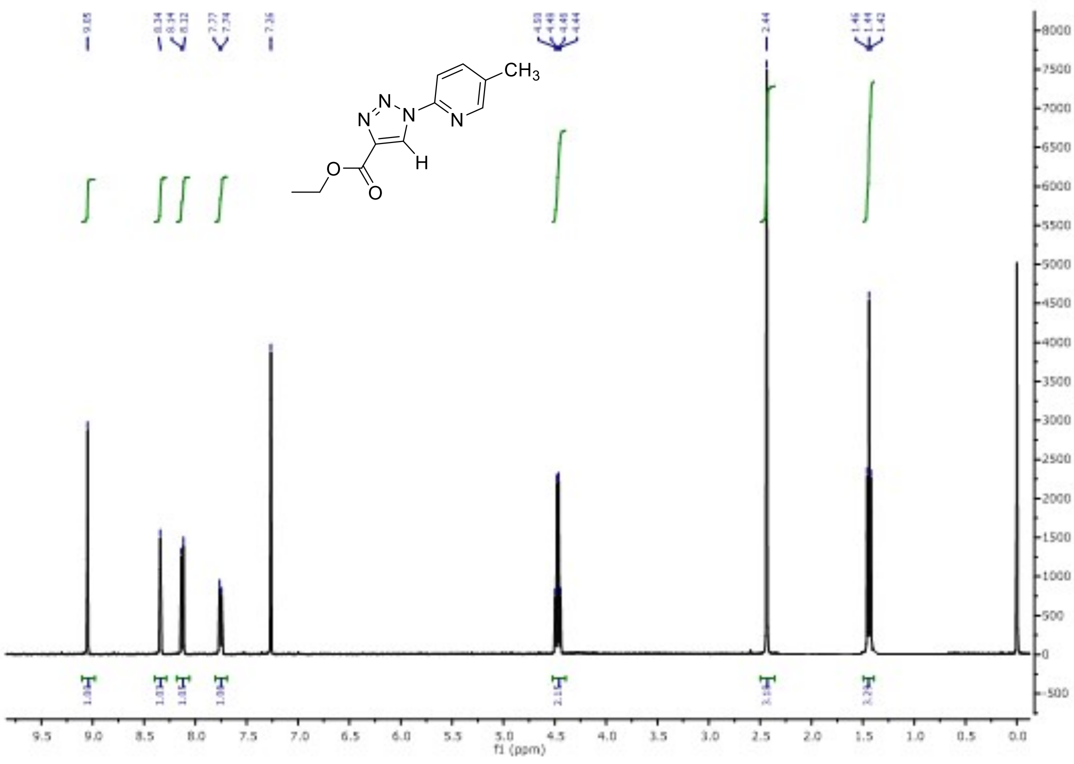

Compound **26d** ^{13}C NMR (101 MHz, $CDCl_3$)

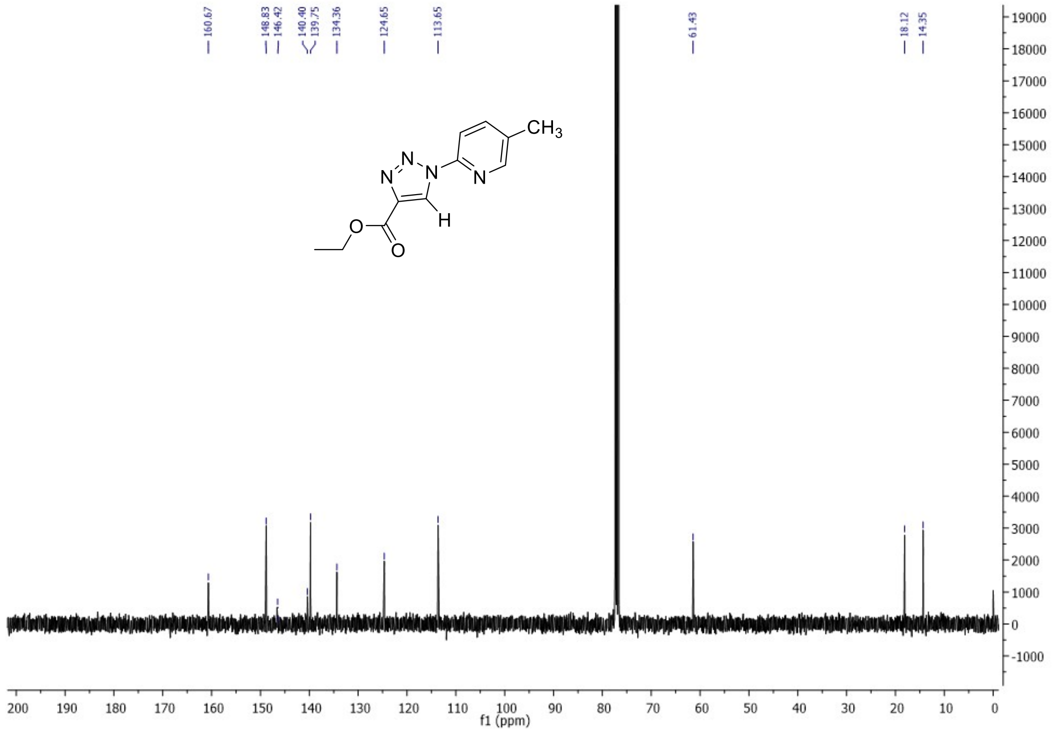

Compound **26e** (400 MHz, $CDCl_3$)

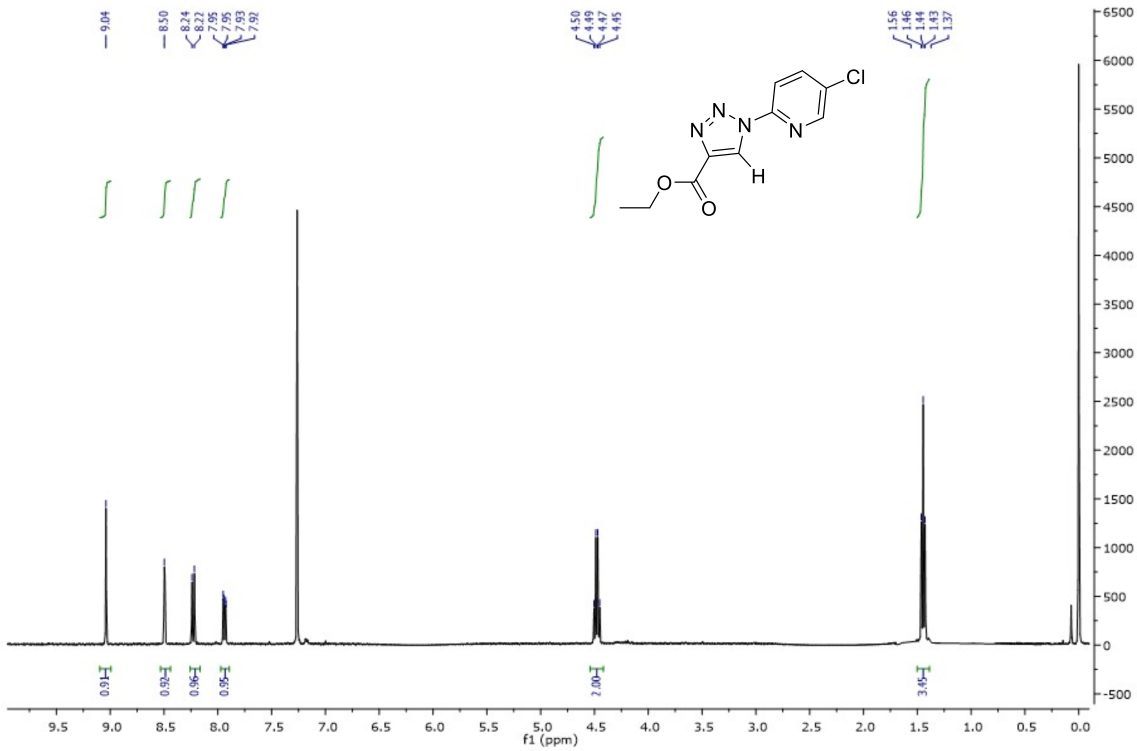

Compound **26e** ^{13}C NMR (101 MHz, $CDCl_3$)

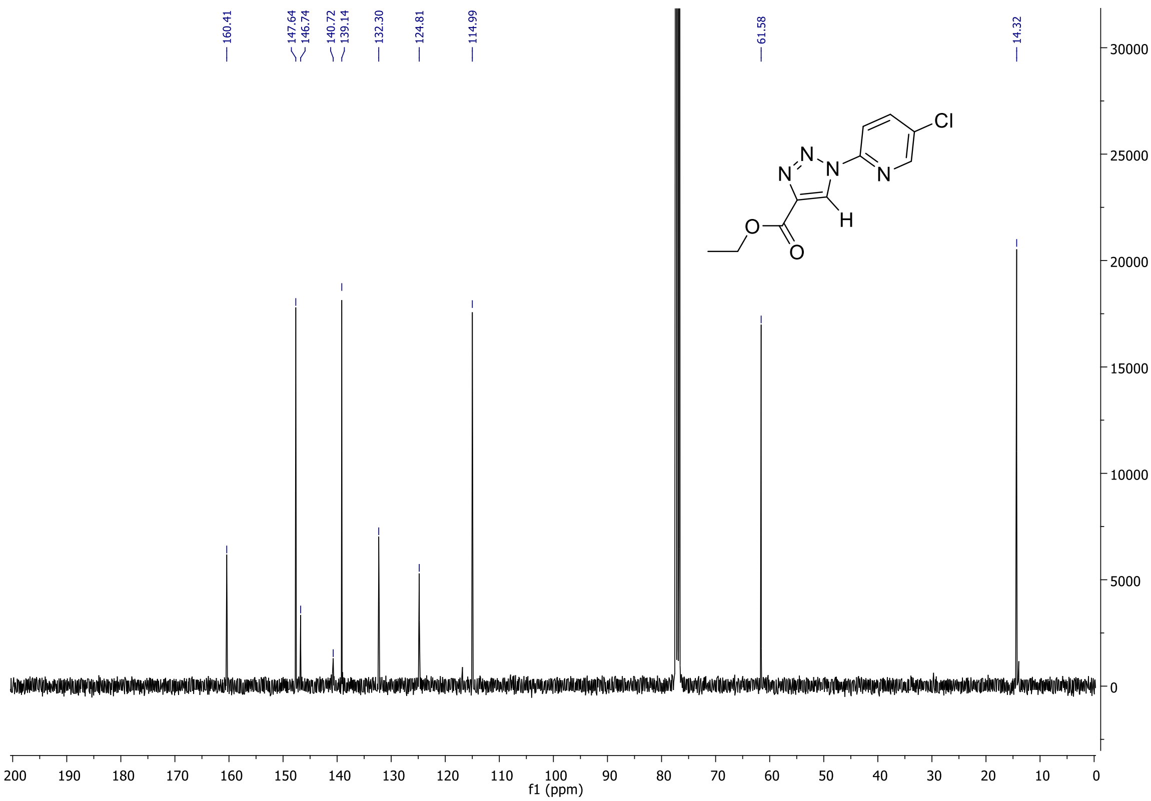

Compound **16** (400 MHz, $CDCl_3$)

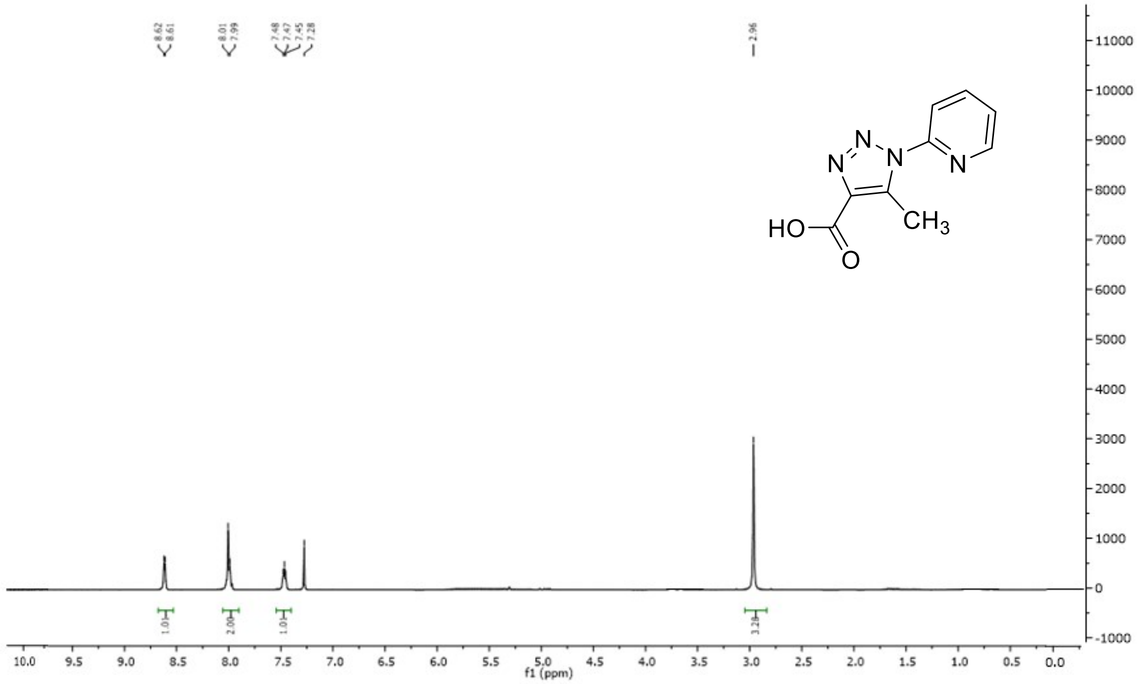

Compound **16** ^{13}C NMR (101 MHz, $CDCl_3$)

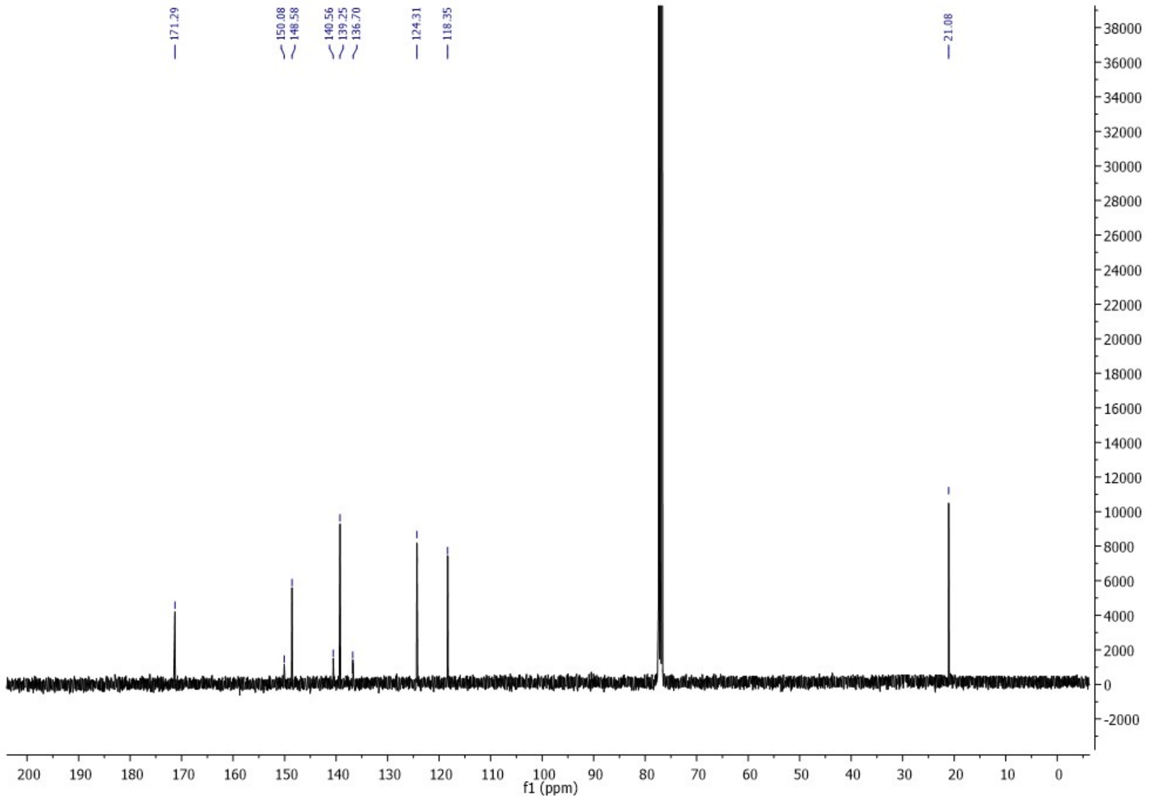

Compound **27a** (400 MHz, CD_3OD)

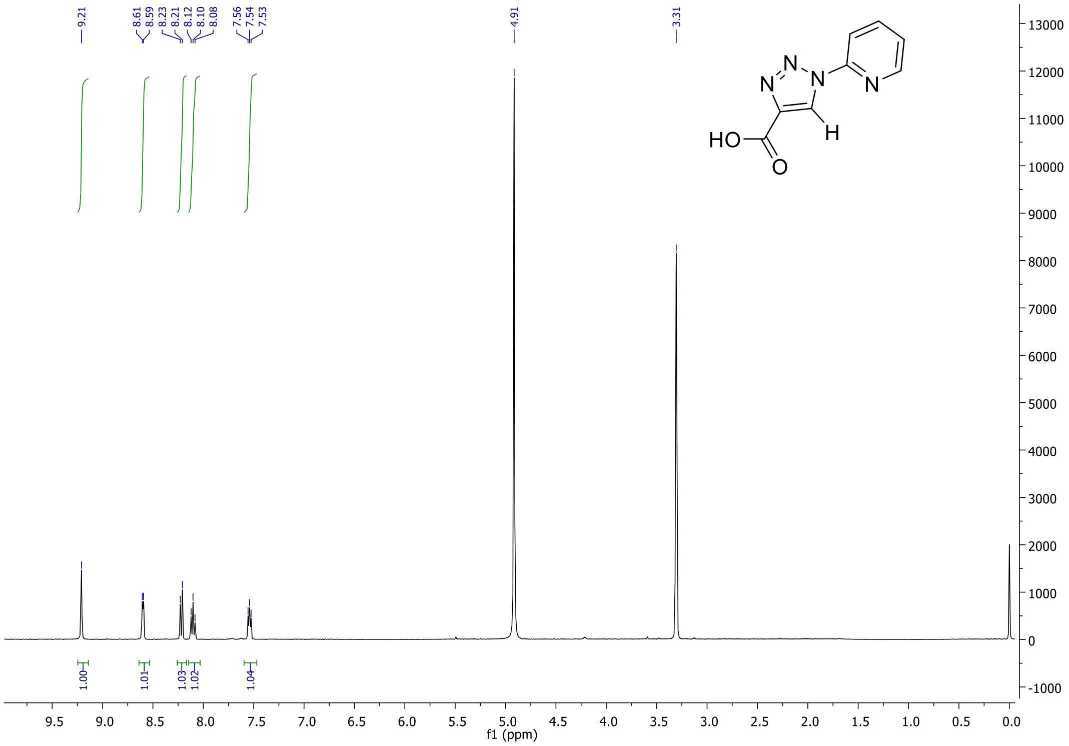

Compound **27a** ^{13}C NMR (101 MHz, CD_3OD)

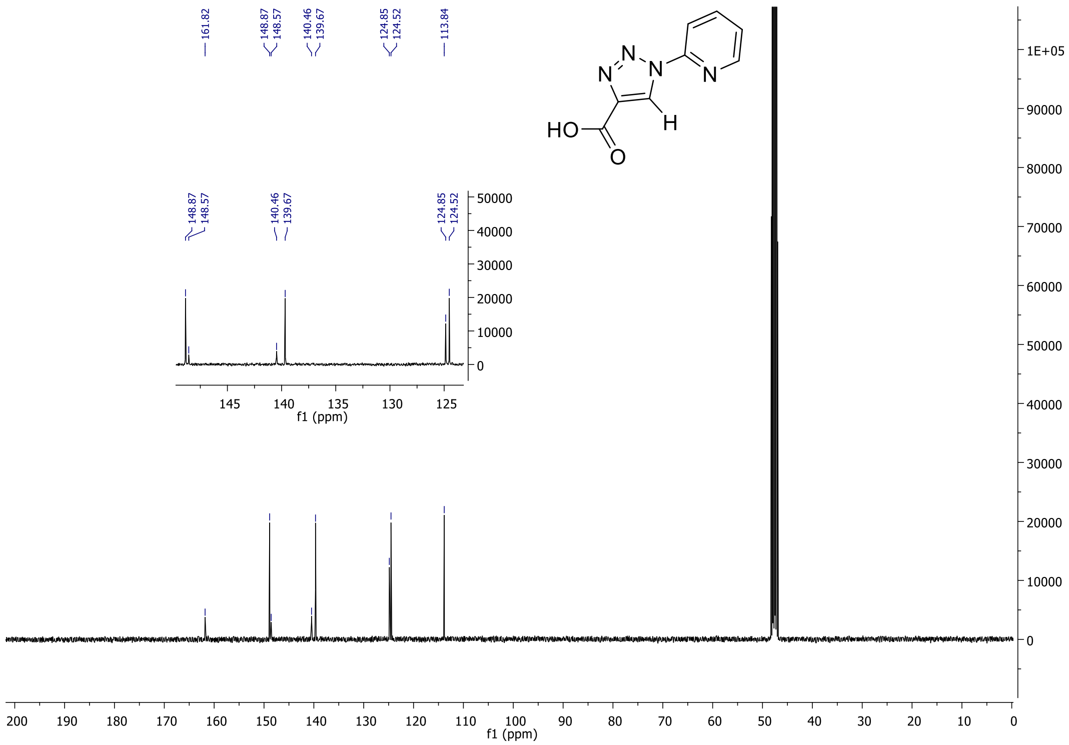

Compound **27b** (400 MHz, CD_3OD)

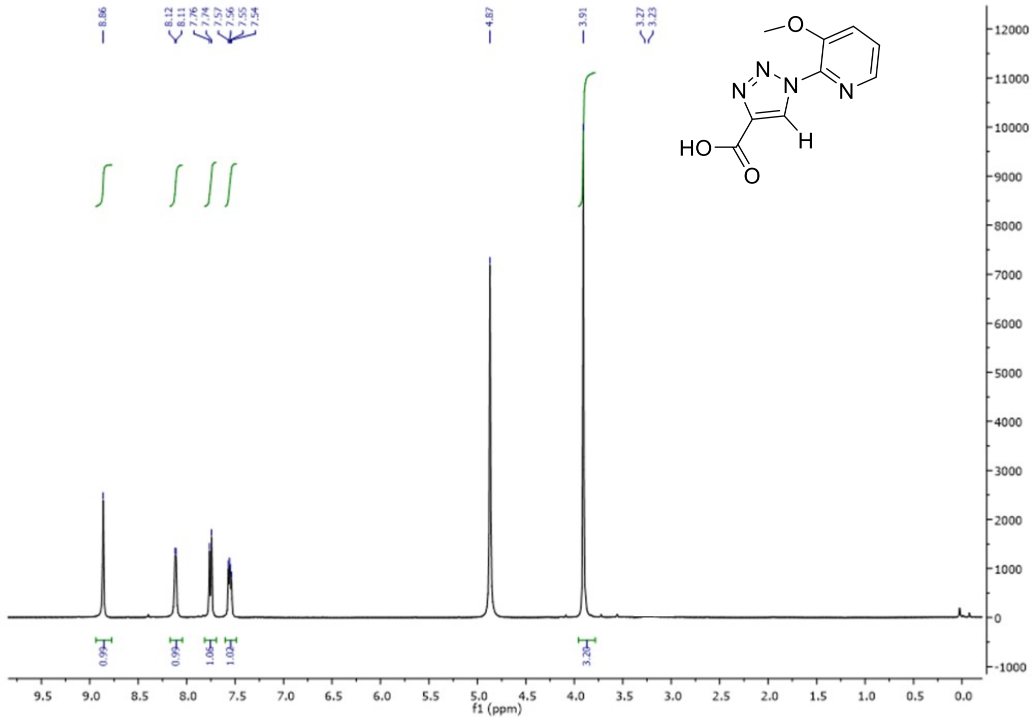

Compound **27b** ^{13}C NMR (101 MHz, CD_3OD)

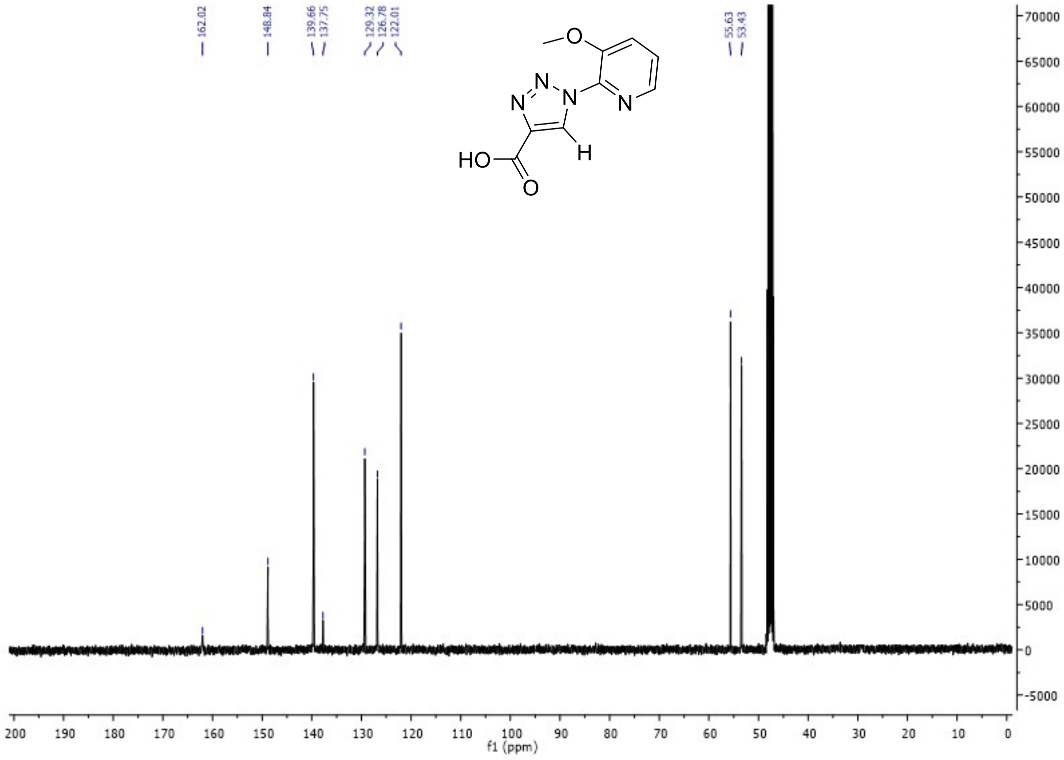

Compound **27c** (400 MHz, CD_3OD)

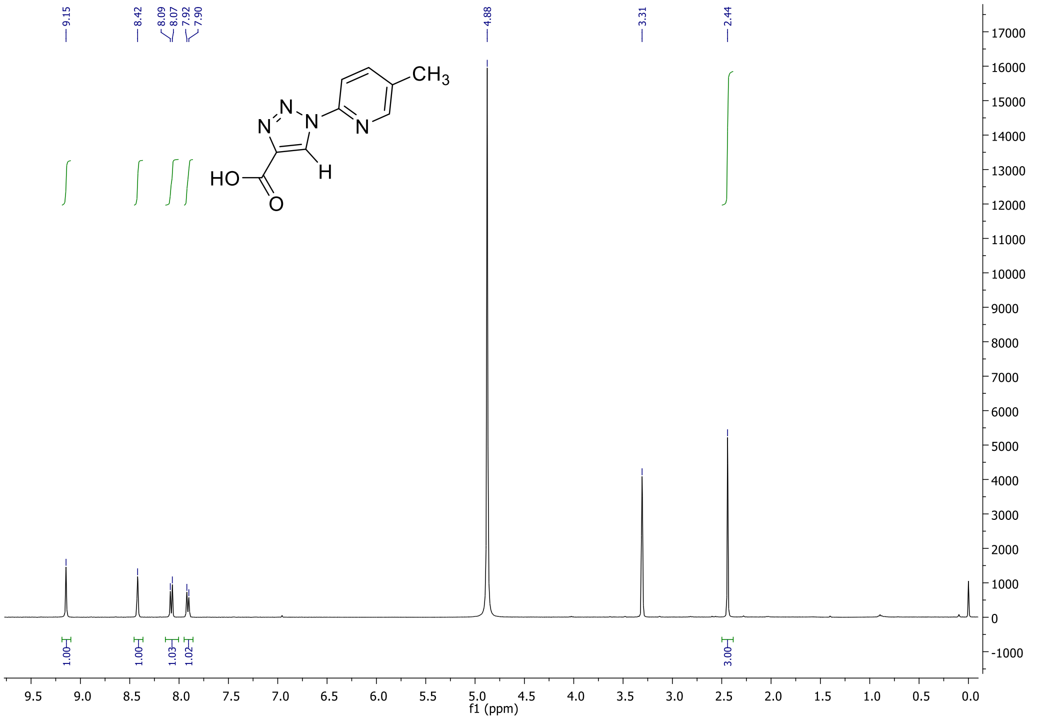

Compound **27c** ^{13}C NMR (101 MHz, CD_3OD)

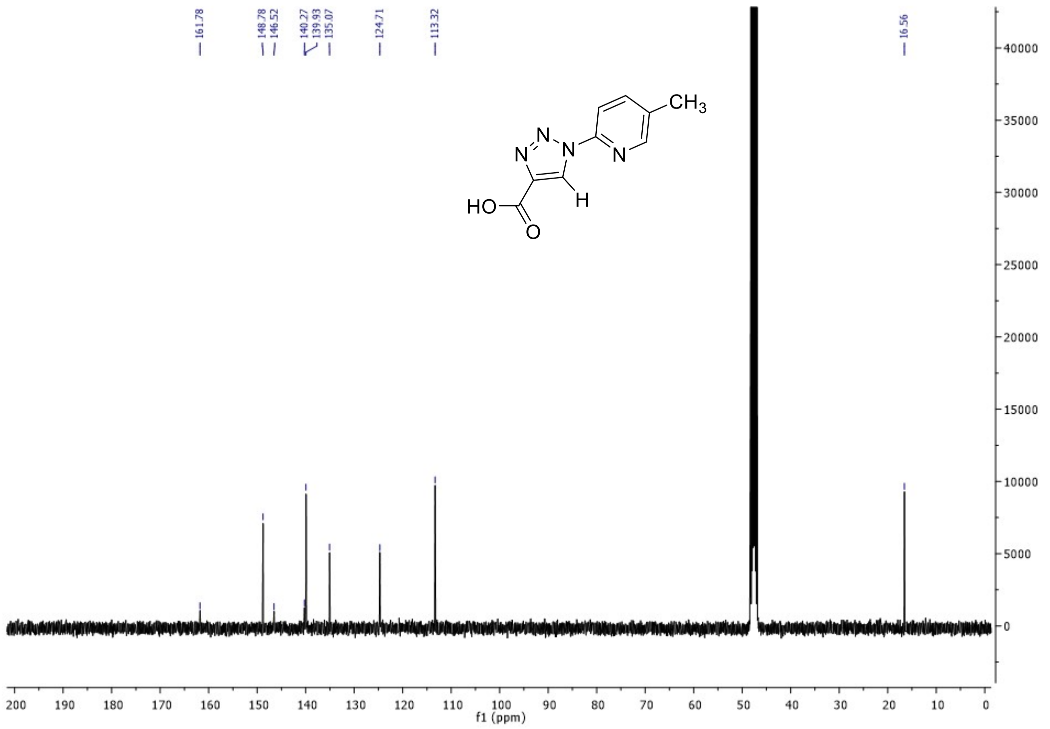

Compound **27d** (400 MHz, CD_3OD)

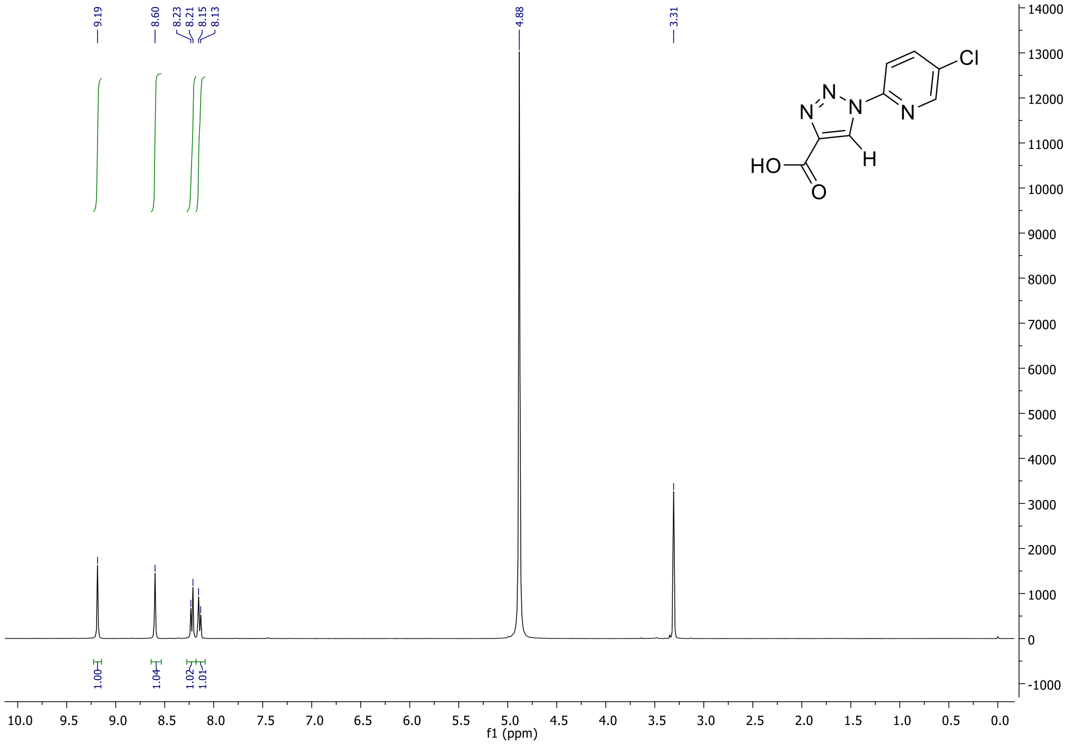

Compound **27d** ^{13}C NMR (101 MHz, CD_3OD)

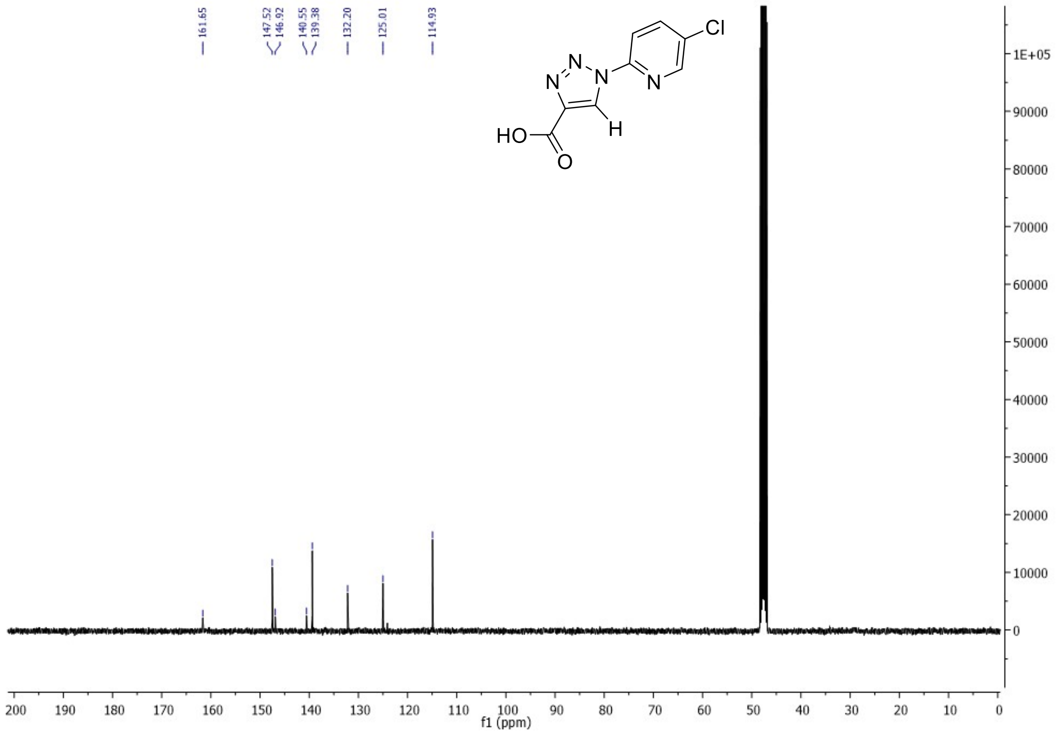

3. *In vitro* tests performed on pyridyl-1,2,3-triazoles 16, 26c and 27a-d in U937 leukemia cell lines.

3.1 Cell cultures

U937 cell line (DMSZ) was cultured in Roswell Park Memorial Institute (RPMI) 1640 Medium supplemented with 10% fetal bovine serum (FBS), 2 mM L-glutamine, and antibiotics (100 U/mL penicillin, 100 μg/mL streptomycin, and 250 ng/mL amphotericin-B). In all the experiments, the cells were treated with compounds **16**, **26c** and **27a-d** at different concentrations and SAHA at a 5μM concentration as positive control of acetylation.

3.2 Western Blot analysis

Cells were lysed in RIPA buffer (1% Triton X-100, 0.1% SDS, 150 mM NaCl, 1 mM EDTA pH 8, 10 mM Tris-HCl pH 8) containing 1% protease inhibitor cocktail. Cell extract was sonicated for 5 minutes (30 seconds ON and 30 seconds OFF) and centrifuged for 15 minutes at 4 °C, the supernatant was then diluted 1:1 in sample buffer 2X Laemmli (0.217 M Tris-HCl pH 8.0, 52.17% SDS, 17.4% glycerol, 0.026% bromo-phenol blue, 8.7% beta-mercapto-ethanol) and subsequently boiled for 3 minutes at 99°C; 15 μg of total protein extract was run and separated by SDS-polyacrylamide gel electrophoresis and then blotted on nitrocellulose membrane (Bio-Rad). Blotted proteins were incubated ON with specific antibodies: anti-acetyl α-tubulin, anti-GADPH, anti-H4 and anti H3K9/14ac. Protein expression was detected by enhanced chemiluminescence method (ECL).

3.3 Histones' extraction

Cells were harvested and washed twice with cold 1X PBS and lysed in Tritonextraction buffer (TEB; PBS containing 0.5% Triton X 100 (v/v), 2 mM PMSF, 0.02% (w/v) NaN_3) at a cell density of 107 cells/mL for 10 minutes on ice, with gentle stirring. After centrifugation (2000 rpm at 4 °C for 10 minutes), the supernatant was removed, and the pellet was washed in half the volume of TEB and centrifuged as before. The pellet was suspended in 0.2 N HCl at a cell density of 4 × 107 cells/mL overnight at 4 °C on a rolling table. The samples were then centrifuged at 2000 rpm for 10 minutes at 4 °C and the supernatant for each of them was collected.

3.4 Cell Proliferation Assay (MTT Assay)

Thiazolyl blue tetrazolium bromide (MTT) assay was carried out as follows. Cells were collected and counted; 30.000 cells were seeded per well in 48-well plate. The cells were treated with compounds **16**, **26c** and **27a-d** at different concentrations and with SAHA and then kept at 37°C in 5% CO_2 for 24 hours. At this point, 50 µL of (5 mg/mL) MTT reagent was added to each well and the plate was placed at 37°C in the incubator for 4 hours. 300 µL of dimethyl sulfoxide (DMSO) was added to each well after aspirating the supernatant. Colored formazan product was assayed spectrophotometrically at 570 nm using TECAN infinite M200 plate reader.

3.5 Enhanced Cell Counting Kit 8 (WST-8/CCK8)

CCK8 was performed according to manufacturer instructions:[9] 100 µL of cell suspension was added per well to the 96 well microplate. Cells were then incubated at 37°C, in a 5% CO2 incubator for 24 hours. At this point, 10 µL of compounds **16**, **26c** and **27a-d** at different concentrations were added to each well and incubated with the cells for 24 hours at 37°C, in a 5% CO2 incubator. After that, 10 µL of CCK-8 Buffer was added and the cells incubated again at 37°C, in a 5% CO2 incubator for 1-4 hours. Absorbance with microplate reader at 450 nm then followed.

4. KAT2A assay performed on triazoles 16, 26c and 27a-d.

The fluorogenic assay for KAT2A potential inhibitors screening of compounds **16**, **26c** and **27a-d** was provided by West Bioscience protocol[10] and set up as follows:

- A positive control, in which the potential inhibitor was excluded from the test.
- An autoacetylation control, without the presence of either the inhibitor or the H3 peptide.
- A negative control, in which the presence of the enzyme was excluded.
- And the proper test for screening the inhibition power of triazoles 16, 26c and 28a-d, with all the components present.

The inhibition test was set up as follows (Table 1): Acetyl CoA (0.2 μM, 80 μL), H3 peptide (0.32 μM, 400 μL), the inhibitors (**16**, **26c** and **27a-d**), dissolved in 1% N-methylpyrrolidone, 20 μL, in a range of four different concentrations (1.5 μM, 5 μM, 10 μM and 15 μM) and the KAT2A enzyme (0.1 μM, 200 μL) were incubated in a water bath at 37°C for 30 minutes in 2 ml volume of TRIS HCL, pH=8 buffer in a glass vial. After incubation with acetyl CoA and the potential inhibitor, KAT2A generated acylated H3 peptide and CoASH. After 30 minutes, the reaction was stopped by the addition of 50 μL of isopropanol (0.8 M) followed by introduction of 100 μL of fluoresceine isothiocyanate isomer I in DMSO (0.4 μM). After 20 additional minutes of incubation at room temperature, the samples were analysed with a fluorescence spectrophotometer to quantify the actual fluorescence emitted (measured at excitation = 495nm and emission = 519nm). The inhibition tests were performed, as per protocol, three times for each compound (Table 1).

Table 1: Set-up of the fluorescence tests performed on triazoles **16**, **26c** and **28a-d** against KAT2A.

	Positive control	Negative control	Autoacetylation Control	Test inhibitor
Buffer	1.52 mL	1.70 mL	1.72 mL	1.50 mL
Acetyl-CoA	80 µL	80 µL	80 µL	80 µL
H3 Peptide	400 µL	400 µL	-	400 µL
KAT2A	200 µL	-	200 µL	200 µL
Test inhibitor	-	20 µL	-	20 µL

5. References

[1] Pagoti, S., Surana, S., Chauhan, A., Parasar, B., Dash, J. (**2013**), Reduction of organic azides to amines using reusable Fe3O4 nanoparticles in aqueous medium., *Catal. Sci. Technol.*, *3*(3), 584–588.

[2] Siragusa, L., Cross, S., Baroni, M., Goracci, L. and Cruciani, G. (**2015**), BioGPS: Navigating biological space to predict polypharmacology, off-targeting, and selectivity. *Proteins*, *83*, 517-532.

[3] Cross, S., Baroni, M., Carosati, E., Benedetti P., and Clementi, S. (**2010**), FLAP: GRID Molecular Interaction Fields in Virtual Screening. Validation using the DUD Data Set, *J. Chem. Inf. Model.*, *50*(8), 1442-1450.

[4] Bajusz, D.; Rácz, A.; Héberger, K. (**2015**), Why Is Tanimoto Index an Appropriate Choice for Fingerprint-Based Similarity Calculations?, *J. Cheminform.*, *7* (1), 1–13

[5] Sabater, S., Müller-Bunz, H., Albrecht, M. (**2016**), Carboxylate-Functionalized Mesoionic Carbene Precursors: Decarboxylation, Ruthenium Bonding, and Catalytic Activity in Hydrogen Transfer Reactions., *Organometallics*, *35*(13), 2256–2266.

[6] Chattopadhyay, B., Vera, C. I. R., Chuprakov, S., Gevorgyan, V. (**2010**), Fused tetrazoles as azide surrogates in click reaction: Efficient synthesis of N-heterocycle-substituted 1,2,3-triazoles., *Org. Lett.*, *12*(9), 2166–2169.

[7] Detrick, F., Toms, R., Forest, W., Alto, P., Richard, J., Place, T.I., (**2018**), WO 2018/160845 Al (51). 12.

[8] Mowbray, C. E.; Braillard, S.; Glossop, P. A.; Whitlock, G. A.; Jacobs, R. T.; Speake, J.; Pandi, B.; Nare, B.; Maes, L.; Yardley, V.; Freund, Y.; Wall, R. J.; Carvalho, S.; Bello, D.; Van Den Kerkhof, M.; Caljon, G.; Gilbert, I. H.; Corpas-Lopez, V.; Lukac, I.; Patterson, S.; Zuccotto, F. (**2021**), DNDI-6148: A Novel Benzoxaborole Preclinical Candidate for the Treatment of Visceral Leishmaniasis. *J. Med. Chem.*, *64* (21), 16159–16176.

[9] https://www.elabscience.com/p-enhanced_cell_counting_kit_8_wst_8_cck8_-388417.html

[10] https://westbioscience.com/acetyltransferase/assay-kit/gcn5-fluorogenic-assay-kit-3372.html
